# Low-detergent sonication decellularization preserves extracellular matrix architecture and enhances cell infiltration in human acellular dermal matrix

**DOI:** 10.3389/fbioe.2026.1839855

**Published:** 2026-08-10

**Authors:** Mzwandile Mbele, Tonétha Jay, Nonhlanhla P. Khumalo, Ardeshir Bayat

**Affiliations:** 1 Hair and Skin Research Laboratory, MRC-SA Wound Healing Unit, Division of Dermatology, Department of Medicine, Faculty of Health Sciences, Groote Schuur Hospital, University of Cape Town, Cape Town, South Africa; 2 Busamed Paardevlei Private Hospital, Cape Town, South Africa

**Keywords:** acellular dermal matrix, cell proliferation, decellularization, extracellular matrix, recellularization, regenerative medicine

## Abstract

**Introduction:**

Acellular dermal matrix (ADM) scaffolds support tissue regeneration by providing structural integrity, biological cues, and a permissive microenvironment; optimal ADMs integrate with native tissue while preserving extracellular matrix (ECM) composition, microarchitecture, and biomechanics. Decellularization is a critical step in ADM development, as incomplete cellular removal can induce inflammation, whereas excessive chemical treatment can disrupt ECM structure and impair scaffold biofunctionality.

**Methods:**

Here, we optimized human dermal decellularization to develop ADMs that retain ECM instruction while enabling deep host-cell infiltration and effective recellularization. Two low-detergent protocols were evaluated, Protocol A (sequential hypotonic and hypertonic solutions) and Protocol B (0.1% Triton X-100 in a hypotonic solution), both incorporating controlled ultrasonication and benzonase treatment, and compared with a conventional high-detergent method (Protocol C).

**Results:**

All protocols effectively removed cellular material and nucleic acids, with substantial reductions in residual DNA content; however, Protocols A and B better preserved key ECM proteins, including collagen IV, elastin, and laminin, as confirmed by Raman spectroscopy and scanning electron microscopy. Functional assays demonstrated enhanced fibroblast adhesion and cell proliferation in Protocols A and B, whereas Protocol C showed limited cell growth. In ex vivo human skin wound models, Protocol B supported the highest cell infiltration and ECM deposition by day 21, followed by Protocol A, while Protocol C exhibited minimal integration and remodeling. Histological and immunohistochemical analyses consistently confirmed superior ECM preservation with low-detergent approaches.

**Conclusions:**

Overall, combining controlled ultrasonication with minimal non-ionic detergent exposure represents a promising decellularization strategy that preserves ECM architecture, supports recellularization, and enhances scaffold integration potential for tissue engineering applications.

## 1 Introduction

Wound healing presents significant challenges in various tissues, including skin, bone, muscle, nerve, internal organs, and mucosal membranes. Chronicity, poor vascularization, and comorbidities such as aging or infection complicate healing (Demidova-Rice et al., 2012; Greaves et al., 2013a; Greaves et al., 2013b; Kolimi et al., 2022; Negut et al., 2020; Okur et al., 2020; Rehnke et al., 2024), while scarring, fibrosis, and impaired cellular function further impede healing, especially in older adults or those with compromised immune systems (Greaves et al., 2013a; Greaves et al., 2013b).

Additional factors, including infection, reduced collagen production, and lifestyle choices (e.g., poor nutrition, smoking), also hinder the wound repair process (Demidova-Rice et al., 2012; Okur et al., 2020). Consequently, advancements in regenerative medicine, tissue engineering, and targeted therapies are essential to improve healing outcomes across different tissue types (Kolimi et al., 2022).

Among regenerative strategies, acellular dermal matrix (ADM) scaffolds have emerged as important biomaterials because they provide structural support, preserve extracellular matrix (ECM) architecture, and promote cellular infiltration and tissue remodeling. In burns and wound healing, scaffolds made from biocompatible materials like collagen and synthetic polymers can prevent wound infection while facilitating tissue regeneration (Negut et al., 2020). Although bioengineered scaffolds are used in multiple surgical contexts, this study focuses on ADM for wound healing (Abdul-Al et al., 2020; Rehnke et al., 2024). Advancements in biomaterials, 3D printing, and nanotechnology continue to improve scaffold design, offering solutions for tissue regeneration and implant therapies (Tang et al., 2025). ADM scaffold development plays a crucial role in regenerative medicine for a variety of applications, including wound healing, burn treatment, repair of tissue defects, and breast implant reconstruction (Crapo et al., 2011; Tang et al., 2025).

The use of skin grafts, including ADM, has increased for the treatment of acute and chronic wounds, particularly deep wounds. Human skin offers several advantages as a source of ADM scaffolds, including favorable integration capacity, support for cell adhesion and proliferation, and reduced risk of immune rejection compared with xenogeneic sources (Crapo et al., 2011). Biocompatible and bioactive biological ADMs are generated through tissue decellularization, a process by which antigenic cellular components are removed while preserving the ECM. ADM is an allograft tissue where the preserved extracellular matrix is processed by eliminating antigenic tissue (cellular) components, while retaining the native tissue ECM structure, composition, mechanical properties, and biological functions (Novotna et al., 2023; Tognetti et al., 2017; Wong and Griffiths, 2014). Due to increasing demand for skin grafts and substitutes, as well as a global donor shortage (Schlottmann et al., 2024; Tognetti et al., 2017), researchers are exploring alternative tissue sources. Decellularization techniques enable the generation of both allogeneic ADM from human cadaveric skin and xenogeneic ADM from animal-derived tissues (Mihalecko et al., 2022).

ADM development utilizes decellularization approaches to remove cells from a variety of tissues with the use of detergents such as sodium dodecyl sulfate (SDS), sodium deoxycholate (SDC), and Triton X-100, enzymatic digestion (e.g., trypsin), and/or osmotic lysis (hypotonic and hypertonic solutions) (Baptista et al., 2011; Sullivan et al., 2012; Zhou et al., 2010). However, commonly used detergents like SDS can disrupt integral ECM proteins due to their denaturing effect and high protein-binding affinity. SDS can intercalate into the collagen triple helix, perturbing collagen structure, reducing ADM collagen content, disrupting the elastin network, and altering ECM functional properties (Lopera Higuita and Griffiths, 2020; Samouillan et al., 2011; Somasekhar and Griffiths, 2023; Wong et al., 2016).

Critically, many high-detergent protocols that achieve excellent cellular clearance *in vitro* may underperform *in vivo* because excessive detergent exposure strips the ECM of bioactive signaling molecules, basement membrane components, and ultrastructural cues required for host-cell migration, integration, and remodeling. This loss of bioactive signaling and structural guidance can translate into poor cellular infiltration, impaired scaffold integration, and suboptimal regenerative outcomes following implantation. Therefore, the key challenge in ADM development is to balance effective decellularization with preservation of ECM structure, composition, and biological function.

Accordingly, the use of low detergent concentrations during skin tissue decellularization is critical to achieving substantial cellular removal while preserving ECM integrity. Ineffective decellularization can induce an inflammatory response, while excessive disruption of ADM ultrastructure, growth factors, and components can negatively impact cytocompatibility and functional tissue replacement (Keane et al., 2012; White et al., 2017).

Using human skin as a scaffold source for tissue engineering offers several advantages, including preservation of native ECM composition, favorable cellular compatibility, and support for tissue regeneration (Chan and Leong, 2008). Human skin contains essential components like collagen and elastin that support cellular adhesion, migration, proliferation, and differentiation, making it ideal for wound healing and tissue repair (Schultz et al., 2011). The native ECM architecture of human skin makes it an attractive substrate for refined decellularization strategies. Preservation of this architecture may enhance cellular integration, reduce scarring, and provide a more physiologically relevant healing environment compared with synthetic or animal-derived scaffolds, with potential applications in burns, chronic wounds, and breast reconstruction (Jin et al., 2025). Therefore, this study aimed to develop and evaluate low-detergent, sonication-assisted decellularization protocols capable of preserving extracellular matrix architecture while improving scaffold permeability, cell infiltration, and tissue remodeling. Human breast skin tissue was decellularized using enzymatic treatment, osmotic lysis approaches, and low concentrations of Triton X-100 to maximize cellular removal while minimizing disruption of ADM ultrastructure and biofunctionality. We hypothesized that coupling controlled ultrasonication with minimal non-ionic detergent exposure would enhance cellular clearance while generating a more permissive microarchitecture for host-cell ingress without compromising biochemical or mechanical integrity.

## 2 Materials and methods

### 2.1 Human breast skin harvesting

Human breast skin was harvested from Groote Schuur Hospital, and Busamed Paardevlei Private Hospital, Cape Town, South Africa, after breast reduction procedures. Breast skin tissue was collected with written informed donor consent and ethical approval from the University of Cape Town, Faculty of Health Sciences, Human Research Ethics Committee (HREC) (REF: 772/2022). All procedures were conducted in accordance with institutional guidelines and ethical standards.

Human breast skin was chosen as the tissue source due to its availability as surgical waste, its similarity to skin from other body regions, and its well-characterized ECM composition. The human tissue used in this study was obtained from excess breast tissue, surgically dissected, and removed by medical professionals during a standard procedure. The tissue was immediately cut into smaller pieces using sterile surgical blades and placed into 50 mL tubes with 20 mL of 30% glycerol solution for temporary preservation. These tubes were kept on ice to preserve tissue integrity. Once collected, they were stored at −20 °C and used within 7 days of collection. Prior to decellularization, tissue samples were thawed at 4 °C and rinsed thoroughly in phosphate-buffered saline (PBS) to remove residual glycerol and debris before further processing.

### 2.2 ADM development

#### 2.2.1 Epidermal removal

Six independent donors were recruited for this study. All three protocols were conducted from each donor and the replicates were performed from different donors to determine if there is any donor-to-donor variability. The age was between 50 and 61 years. Skin sections (approximately 3 × 3 cm^2^, 1 mm thick) were washed with 1× PBS and incubated in 70% ethanol for 24 h to remove lipids and assist epidermal separation. Post-incubation, samples were washed twice with 1× PBS for 20 min each on an orbital shaker and transferred to a hypertonic solution (10 mM Tris-HCl, 1 M NaCl, 10 mM EDTA) at 37 °C for 4–6 h to facilitate epidermal separation. The dermis was then washed twice with 1× PBS for 20 min each.

#### 2.2.2 Acellular scaffold development

Three decellularization protocols were evaluated to determine optimal ADM generation conditions. Six independent donors were recruited. Tissue from each donor was subdivided where sufficient material was available and processed using Protocols A, B, and C. For quantitative analyses, three independent biological replicates per protocol were analyzed unless otherwise stated, with technical triplicates performed where applicable. Protocols A and B were newly developed low-detergent approaches designed to preserve ECM integrity while enhancing cellular removal, whereas Protocol C represented a modified high-detergent protocol adapted from Milan et al. (2020) with adjustments for tissue thickness and sample size.

For Protocol A, after sonication, samples were placed on an orbital shaker at 4 °C, shaking at 150 rpm for 24 h. Every 6 h, the hypotonic (10 mM Tris; 5 mM EDTA) solution was changed. The following day, tissue samples were washed twice for 20 min each with 1× phosphate-buffered saline containing penicillin/streptomycin (PBS-P/S). They were then transferred into a hypotonic solution for another 24 h with 6-hourly solution changes.

To further achieve decellularization in Protocol B, skin samples were subjected to the treatment as mentioned above. The dermal tissue was placed in 0.1% Triton X-100 in hypotonic (10 mM Tris; 5 mM EDTA) solution that was also changed every 6-h period and incubated at 37 °C in a shaking incubator (150 rpm). Then both treated tissues from Protocols A and B were treated with 0.1% Benzonase® endonuclease (E8263 -5KU, Sigma-Aldrich) diluted in PBS (pH 7.4) at 37 °C for 3 h. After 3 h the tissue was introduced to 0.5% EDTA in 1× PBS to neutralize the nuclease activity for 3 h.

Protocol C was adapted from Milan et al. (2020) with modifications for sample size. After epidermal removal, dermal samples were incubated in 1% Triton X-100 in hypotonic buffer (10 mM Tris, 5 mM EDTA) for 48 h (solution changed every 12 h), followed by 0.5% SDS in the same hypotonic buffer for 48 h (solution changed every 12 h). Incubations were performed at 37 °C with orbital shaking at 150 rpm. Samples then underwent identical benzonase treatment (0.1% Benzonase® in PBS, pH 7.4, 3 h at 37 °C) and EDTA neutralization (0.5% EDTA in PBS, 3 h at 37 °C) as described for Protocols A and B.

### 2.3 Validation of developed acellular scaffolds

#### 2.3.1 DNA extraction and quantification

Tissue and ADM samples were obtained using a 4 mm biopsy puncher, with each sample weighing between 18 and 35 mg. Experiments were performed using three independent biological samples per group (n = 3), with technical triplicate measurements conducted where applicable to assess analytical reproducibility. The DNA extraction was performed with the addition of 500–800 µL of cold TRIzol™ reagent (Ambion, Life Technologies) added to the prepared ADM samples. This step was followed by homogenization utilizing the Omni Bead Ruptor 4 Elite homogenizer (Omni International Inc.) for four cycles, where each cycle consisted of 1 min homogenization followed by 1 min of cooling on ice. The samples were then centrifuged at 12,000 × g for 5 min at 4 °C. The supernatant was transferred to a new tube. A total of 100 µL chloroform (Sigma-Aldrich) was added into the samples and the samples were incubated for 5 min at room temperature. To separate the phases the samples were then centrifuged at 12,000 × g for 15 min at 4 °C and following phase separation, DNA was recovered from the interphase and organic phase according to the manufacturer’s TRIzol protocol. Then 300 µL of 100% ethanol was added to the DNA-containing mixture and gently inverted 20 times. The samples were centrifuged at 2,000 × g for 5 min at 4 °C after incubating for 2 min at room temperature, and the supernatant was discarded. The DNA pellets were resuspended in 1000 µL of 75% ethanol, which was incubated for 15 min with occasional mixing. This was followed by centrifugation at 2,000 × g for 5 min at 4 °C, and the supernatant was discarded. The DNA pellets were air-dried on top of a paper towel for 5–10 min and eluted in 30 µL of elution buffer (Qiagen). DNA concentration was quantified using 1 µL of eluted sample analyzed on a BioDrop µLite spectrophotometer (Scientific Laboratory Supplies).

#### 2.3.2 Statistical analysis

Established decellularization criteria were applied in this study, including residual DNA content below 50 ng/mg dry tissue weight, DNA fragment lengths below 200 base pairs, and absence of visible nuclear material on histological assessment (Crapo et al., 2011).

Residual DNA content was quantified and expressed as ng DNA per mg of dry tissue weight (ng/mg). Measurements were obtained from three independent biological samples per group (n = 3) and are presented as mean ± standard deviation (SD). To calculate the DNA content (ng/mg), this formula was used.

$$\text{DNA content}=\frac{\text{DNA}\;\text{concentration}\;\left(\text{ng}/µL\right)\times \text{Total}\;\text{elution}\;\text{volume}\;\left(µL\right)}{\text{Dry}\;\text{tissue}\;\text{weight}\;\left(\text{mg}\right)}$$

Statistical comparisons between native and decellularized groups (e.g., Protocol A, Protocol B, and Protocol C) were performed using one-way analysis of variance (ANOVA) followed by Tukey’s multiple comparisons *post hoc* test. Where only two groups were compared, an unpaired Student’s t-test was applied.

A p-value <0.05 was considered statistically significant. All analyses were conducted using GraphPad Prism software.

#### 2.3.3 Tissue preparation for histology experiments

Histology experiments were prepared by punching ADM using 4 mm biopsy punches, with the procedure repeated three times (n = 3). Initially, the biopsy tissues were fixed in 10% formalin (Sigma-Aldrich) for 24 h then they were transferred to the tissue processor (Leica, TP1020, serial no: 3356/02.2008). To prepare the fixed tissue samples for microscopic observation, they were embedded in a solid paraffin wax medium. The process involves the following steps: fixation, dehydration, clearing, and staining. After tissue processing, the samples were embedded to form tissue blocks using the embedding station (Leica EG1160). The tissue was dehydrated in a series of graded ethanol solutions (70%, 80%, 90%, 95%, and 100%), cleared in xylene, and infiltrated with paraffin wax. After solidification, the tissue blocks were placed in ice-cold water for approximately 1 h. The tissue was then sectioned to 4 µm thickness using a Microtome (Leica, RM2255) and the sections were placed onto enhanced coating hydrophilic slides (Cell Path Services). The tissue sections were air-dried for 1 h before the wax was melted at 60 °C in an incubator for an additional 30–60 min.

#### 2.3.4 Haematoxylin and eosin (H&E) staining

H&E staining was performed to confirm cellular removal through assessment of nuclear absence following decellularization, and ADM architecture after decellularization in comparison with untreated tissue. Sections were cleared and deparaffinized through xylene (2 × 4 minutes), rehydrated in 100% ethanol (2 × 4 minutes), 95% ethanol (2 × 3 minutes), 70% ethanol (1 × 3 minutes), and deionized water (1 × 3 minutes). The sections were then stained in Haematoxylin (1 × 10 min), followed by incubation in Scott’s water (1 × 1 minute) and then incubated in eosin (1 × 5 minutes); in between, the sections were rinsed in deionized water between the steps. Then the sections were dehydrated, and slides were coverslipped using Xylene-based Permount™. The slides were dried at room temperature overnight. The H&E staining results were viewed and scanned using a Euromex microscope (Holland) with CMEX Camera 5.0 (sn: KCLL04021279).

#### 2.3.5 Immunohistochemistry (IHC) and protein signal analysis

To confirm the conservation of key ECM proteins of interest (collagen I, III, and IV, elastin, and laminin), IHC was performed across all methods (A-C). The following primary antibodies were used: collagen I (Abcam, ab34710), collagen III (Abcam, ab7778), collagen IV (Abcam, ab6586), elastin (Abcam, ab21610), and laminin (Abcam, ab9519). Tissue sections were cleared and deparaffinized in xylene for 2 × 4 minutes, followed by rehydration in 100% ethanol (2 × 4 minutes), 95% ethanol (2 × 3 minutes), 70% ethanol (1 × 3 minutes), and deionized water (1 × 3 minutes). The sections were then washed in 1× PBS for 2 × 5 minutes. After the washing step, antigen retrieval was done, sections were immersed in a boiled antigen retrieval solution, sodium citrate buffer (pH 6.0), and allowed to cool down with them for 20 min. Then, endogenous peroxidase activity was blocked in these sections by treating them with 3% hydrogen peroxide for 30 min, and then they were washed in 1× PBS for 2 × 5 minutes. The sections were subjected to 1% BSA for 30 min to block non-specific antibody binding. This was immediately followed by primary antibody addition to the sections, where the incubation took 1 h at room temperature, after which the sections were placed in a 4 °C walk-in refrigerator overnight. The following day, the sections were washed in 1× PBS (2 × 5 minutes). The secondary antibody was then added and incubated at room temperature for 30–60 min, depending on the primary antibody manufacturer’s recommendations. This step was immediately followed by the dehydration, and the slides were coverslipped using Xylene-based Permount™. The slides were left to dry at room temperature overnight. The optimal antibody concentrations were as follows: collagen I (1:200), collagen III (1:100), collagen IV (1:400), elastin (1:100), and laminin (1:400). The immunohistochemistry staining results were examined and scanned using a Euromex microscope (Holland) equipped with a CMEX Camera 5.0 (SN: KCLL04021279). Although antibodies for collagens I and III were initially evaluated, signal specificity was suboptimal during optimization and these markers were therefore excluded from downstream quantitative analyses.

#### 2.3.6 Masson’s trichrome and elastic fiber staining

Masson’s trichrome and elastic fiber staining were performed to analyze collagens and elastic fibers in ADM samples, with direct comparison to untreated skin control samples. The staining protocols were performed according to the manufacturer’s instructions (Abcam, ab150686 and ab150687). Both untreated and ADM sections were cleared and deparaffinized and rehydrated by placing them in xylene (2 × 4 minutes), 100% ethanol (2 × 4 minutes), 95% ethanol (2 × 3 minutes), 70% ethanol (1 × 3 minutes), and deionized water (1 × 3 minutes). The sections were incubated in preheated Bouin’s solution for 15 min and cooled in tap water (18 °C–26 °C). The sections were washed in running water for 1 min.

Next, the sections were stained with Weigert’s Iron Haematoxylin solution for 5 min, followed by Biebrich Scarlet-Acid Fuchsin for 5 min rinsing the sections in between with deionized water. The sections were stained with phosphotungstic acid solution and then transferred directly to aniline blue solution for 5 min each. Finally, the slides were treated with 1% acetic acid for 2 min and rinsed in tap water. The sections were dehydrated through a series of graded ethanols, cleared and deparaffinized in xylene, and mounted with Xylene-based Permount™. The sections were incubated at room temperature overnight to dry the coverslips.

To achieve elastic fiber staining, after deparaffinizing the sections, the sections were immersed in Verhoeff’s Haematoxylin solution for 30 min. The sections were rinsed in tap water. They were then differentiated in 2% ferric chloride solution until the elastic fibers appeared black in color. The sections were washed in water and incubated in sodium thiosulfate to destain any residual iodine. Lastly, the sections were transferred to Van Gieson’s solution. The sections were dehydrated through ethanol, cleared with xylene, mounted with Xylene-based Permount™, and coverslipped. The slides were dried at room temperature overnight. The detailed staining procedures are described in the manufacturer’s protocols.

#### 2.3.7 Localization and quantification of ECM antibody proteins

IHC antibody signals, Masson Trichrome, and elastic staining were localized and analyzed using Fiji ImageJ software (https://imagej.net/software/fiji/downloads following the detailed protocol by Schindelin et al. (2012). The “Color Deconvolution” plugin was used to isolate staining signals, and the *Measure* function was used to quantify area and mean gray value. Images were opened in ImageJ software, and the appropriate color deconvolution method for each image was selected. Image analysis was performed using Fiji ImageJ software. Color deconvolution was used to isolate staining signals, and quantitative measurements of stained area and mean gray value were obtained following standardized thresholding across all groups within each analysis batch. Analyses were performed on comparable regions of interest (ROIs) across samples, with measurements conducted in triplicate (n = 3). Statistical comparisons were performed using one-way ANOVA with Tukey’s *post hoc* test.

#### 2.3.8 Raman spectroscopy analysis

Raman spectroscopy is a technique to examine the molecular/chemical composition of tissue samples. In this process, sections underwent a series of deparaffinization and rehydration steps. The Raman spectroscopy settings were as follows: laser wavelength: 532 nm, laser power: 10 mW, acquisition time: 10 s, to prepare them for analysis. Initially, the slides were immersed in xylene for two sessions of 4 min each to remove the paraffin. The slides were then rehydrated through a series of ethanol solutions: two sessions of 4 min each in 100% ethanol, followed by two sessions of 3 min each in 95% ethanol, and one session of 3 min in 70% ethanol. After the ethanol treatments, the slides were rinsed in deionized water for 3 min to complete the rehydration process. Once these preparation steps were completed, the slides were sent to the Electron Microscope Unit (EMU) in the Materials Engineering Department at the University of Cape Town, South Africa, for Raman spectroscopy analysis using a Tescan MIRA3 RISE SEM platform (Tescan USA). The specific Raman bands analyzed for each protein were as follows: collagen (amide III, 1245 cm-1), elastin (amide I, 1665 cm-1), and laminin (amide I, 1655 cm-1).

The tissue sections were illuminated with a laser, and the scattered light was collected to generate Raman spectra, which provided detailed information about the chemical and structural components of the tissue. The analysis of Raman spectra allowed for the distinction of chemical group variations in tissue samples treated with different protocols, providing insights into the relative protein levels and tissue composition. Characteristic Raman bands associated with collagen, elastin, and laminin were analyzed, including amide I, II, and III spectral regions. At position 1639–1644 cm^-1^ (-C=O stretching), Amide II represented elastin at position 1670–1570 cm^-1^ (-NH_2_ and-N-H deformation), and amide III represented collagen at position 1172–1350 cm^-1^ (-CH and -CN stretching, and -CNH deformation), as illustrated in Table 1.

**TABLE 1:** Characteristic Raman spectral peaks of key ECM proteins.

Wavenumber (cm^-1^)	Collagen	Elastin	Laminin	Assignment/Interpretation
1639–1644 cm	​	X	X	Amide I (-C=O stretching)
1670-1570	​	X	​	Amide II (-NH2 &N-H deformation)
1172-1350	X	​	​	Amide III (-CH and -CN stretching, and -CNH deformation)

#### 2.3.9 Ultra-structural assessment *via* scanning electron microscopy (SEM)

To compare ADM scaffolds with untreated skin, SEM was utilized. To prepare tissue samples for SEM, they were initially fixed using 2.5% (v/v) glutaraldehyde diluted in 1× PBS for an overnight duration. This was followed by a dehydration of these samples through graded ethanol concentrations (30%, 50%, 70%, 80%, 90%, 95%, and 100%), and in each concentration, the samples were incubated for 5 min. Then, the samples were attached to 8 mm aluminum stubs and coated with gold. The samples were then scanned using an FEI NovaNano SEM (LabX, USA) to take high-resolution micrographs that allowed for a detailed examination of the structural variations between untreated skin and ADM human scaffolds. Image analysis was carried out using DigiMizer software to measure the width of the matrix fibers. These measurements were compiled and presented in a table to facilitate a comparative analysis between the normal skin and decellularized human matrix samples.

#### 2.3.10 Mechanical properties evaluation using a universal tensile tester

The mechanical properties of normal skin (NS) and ADM were assessed using a universal tensile testing machine (Instron 5544, Norwood, USA) equipped with a 500N load cell. The evaluation measured the maximum stress of the samples before and after decellularization (DCL) treatment. The parameters that were measured in these samples were the ultimate tensile strength (UTS), maximum strain at failure, and elastic modulus. Samples were secured using pneumatic grips with a gauge length of 20 mm and tested at an extension rate of 10 mm/min until failure.

Untreated and ADM scaffolds were cut into strips of 5 cm length, with 1 cm thickness and 2 mm width measured as the average of three measurements (n = 3). The samples were clamped between two grips. The distance between the grips was adjusted and an extension rate of 10 mm/min was used to pull these samples until failure.

### 2.4 ADM functionality

#### 2.4.1 Preparation and culturing of ADM

Two different cell types, bone marrow-derived cells (DMB), and FG0 fibroblasts, were used to recellularize ADM. The ADM was cut into 8 mm discs using a stainless-steel eyelet hole puncher. The sterilized ADM scaffolds were introduced into 24-well plates with the dermal side facing upward.

Each disc was seeded with DMB, or FG0 cells at a density of 2.5 × 10^2^ cells per disc and incubated at 37 °C for 2 h to allow cell adhesion to the scaffold. The cells were cultured in Dulbecco’s Modified Eagle Medium (DMEM) supplemented with 10% fetal bovine serum (FBS) and 1% penicillin/streptomycin. After the adhesion period, 2 mL of freshly prepared complete culture medium was added to each well. The culture medium was replaced every 3 days, and the experiment was maintained for 21 days.

#### 2.4.2 ADM functionality and cell proliferation

The functionality of the ADM was assessed using two commercially available, ready-to-use assays: WST-1 and PrestoBlue (Abcam, UK). The assays were performed according to the manufacturer’s instructions. These assays were employed to evaluate cell viability and proliferation, key indicators of cell viability, proliferation, and metabolic activity (as a surrogate of scaffold-supported cellular functionality) within a defined cell culture or engineered substitute. This assessment is crucial for suggesting that the generated ADM supports cell functionality.

WST-1 and PrestoBlue assays (Thermo Fisher Scientific, USA) were the two commercially available assays used in this study and performed according to the company’s instructions. For the WST-1 assay, in control (96-well plate surface) and ADM media at day 7, 14, and 21, 100 µL volume of cells and medium +10 µL WST-1 reagent were measured. In PrestoBlue, 90 µL volume of cells and medium +10 µL PrestoBlue reagent was added into a 96-well microplate and incubated for 30–40 min for WST-1 and 1 h for PrestoBlue assays. After incubation, WST-1 absorbance was read at 540 nm wavelength, while the PrestoBlue fluorescence was read at excitation and emission wavelengths at 540 and 590 nm, respectively. Where ADM discs were used, the assay supernatant was transferred to a 96-well plate for optical/fluorescence reading to avoid scattering artifacts from the scaffold.

#### 2.4.3 *Ex vivo* model and acellular scaffold integration capacity

A full-thickness *ex vivo* skin model was utilized to assess scaffold cellular integration and remodelling capacity as well as cellular interaction with fresh human tissue. Excess adipose tissue was trimmed from fresh skin samples prior to processing. Then punched with 8 mm circular puncher (stainless steel eyelet punch). To create standardized artificial wounds, 4 mm punch biopsies were taken from the center of each 8 mm skin disc. The prepared skin models were placed into inserts in a 24-well plate, and 4 mm ADM biopsies were positioned within the central wound areas. Williams E Medium was added to each well to maintain culture conditions, ensuring that the epidermal layer of the *ex-vivo* tissue remained air-exposed to mimic *in-vivo* conditions. The models were monitored daily, and the culture medium was replaced every 3 days to maintain proper nourishment. ADM biopsies were harvested at 0, 7, 14, and 21 days for further experimental analyses, such as assessing cell proliferation and other factors related to wound healing and integration capacity.

#### 2.4.4 *Ex Vivo* model: histology of ECM antibody and total collagen proteins

Histology experiments for the *ex vivo* model were performed on biopsy sections collected at 7, 14, and 21 days. These experiments followed the protocols previously described for H&E staining, IHC, and Masson Trichrome staining. The corresponding methods are detailed in Sections 2.3.3-2.3.5.

#### 2.4.5 Localization and quantification of ECM antibody and total collagen proteins

H&E staining, IHC antibody signals, and Masson’s Trichrome images were analyzed using Fiji ImageJ software (https://imagej.net/software/fiji/downloads), following the detailed protocol described by Schindelin et al. (2012) (Schindelin et al., 2012). Bar graphs and statistical analyses were generated in GraphPad Prism 10. A one-way ANOVA with Tukey’s *post hoc* test was used to compare differences among groups. For each stain, identical thresholding settings were applied across groups within a given analysis batch, and measurements were performed on comparable regions of interest (ROIs) across all samples.

## 3 Results

### 3.1 Nuclei (cells) and DNA detections

**FIGURE 1:**
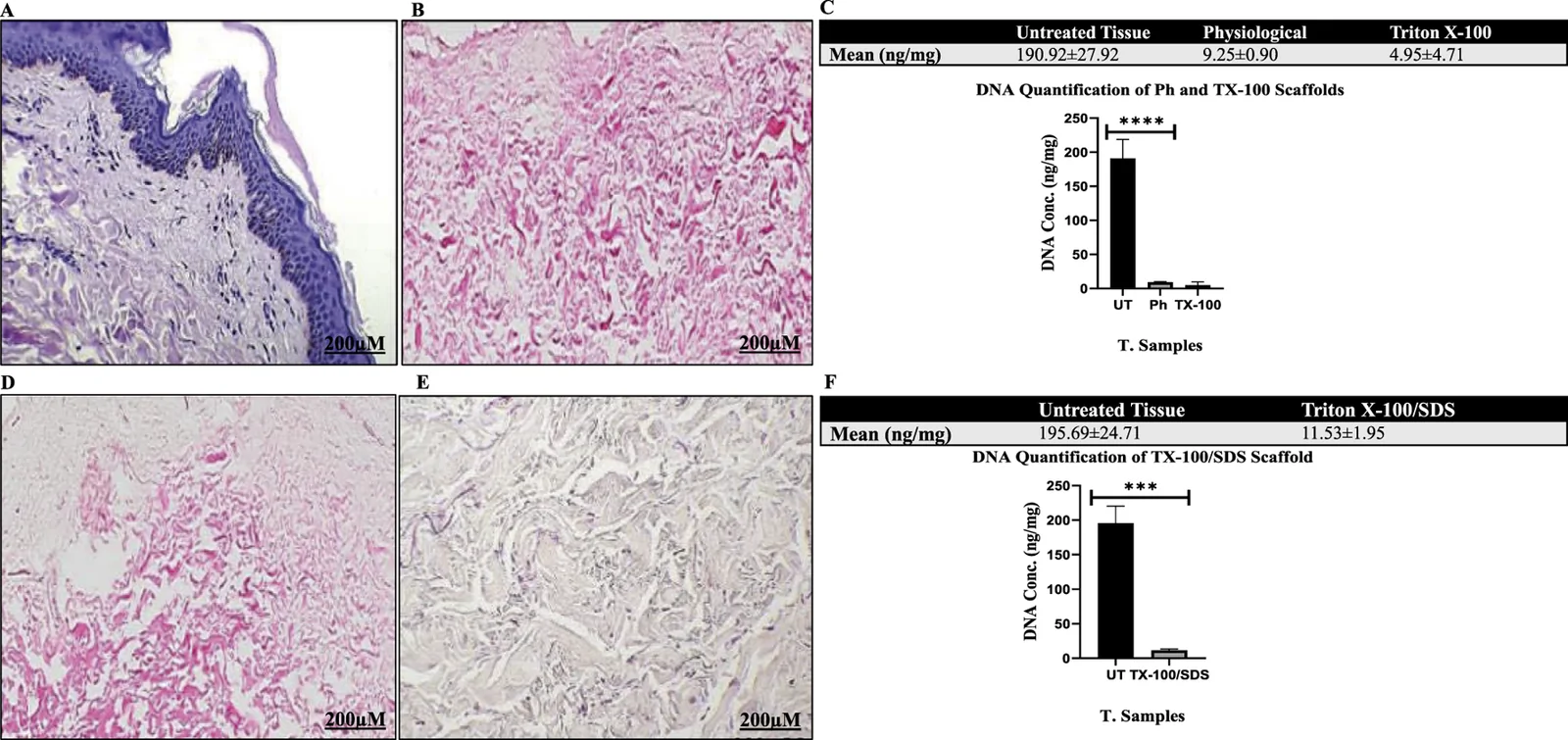
Haematoxylin and Eosin (H&E) staining and DNA quantification of untreated tissue and scaffolds generated using Protocols A–C. **(A)** Untreated tissue showing preserved tissue architecture and nuclei stained blue; **(B)** Protocol A scaffold; **(C)** Protocol B scaffold; and **(D)** Protocol C scaffold. **(E)** DNA content in untreated tissue and scaffolds generated using Protocols A and B. **(F)** DNA content in untreated tissue and scaffolds generated using Protocol C. DNA content is expressed as ng DNA per mg dry tissue weight. Each protocol was performed three times (n = 3); data are presented as mean ± SD. ***p < 0.001 and ****p < 0.0001.

In this study, two novel ADM protocols (A and B) were developed and compared with a published method (Protocol C) to evaluate decellularization efficiency and ECM preservation. H&E staining demonstrated substantial cellular removal, as indicated by the absence of visible nuclei in the ADM groups (Figures 1B-D) compared to the untreated skin tissue (Figure 1A). No residual nuclear material was detected in any ADM group, indicating effective cellular removal.

DNA quantification was performed using biochemical DNA extraction followed by concentration measurement. Quantitative analysis and statistical evaluation were performed using GraphPad Prism software. The data showed a substantial reduction in DNA content across all ADM scaffolds (Figures 1A–F). All protocols demonstrated greater than 90% reduction in DNA content relative to untreated tissue (p < 0.05, one-way ANOVA). No statistically significant differences were observed between the decellularization protocols under the current analytical conditions. These findings suggest substantial cellular removal across all protocols under the current analytical conditions, although interpretation should be considered in light of the absorbance-based DNA quantification approach and absence of DNA fragmentation analysis. All DNA results, including concentration and calculated DNA content, are summarized in Table 2 for Protocols A and B, while Protocol C is presented separately in Table 3 for clarity and comparison. The DNA quantification results have indicated variability among different donors, especially in untreated tissues and decreased in ADM scaffold, these were illustrated by standard deviation that was identified from DNA quantification results. DNA quantification indicated variability among donors, particularly in untreated tissues. This variability was lower in the ADM groups, as reflected by the corresponding standard deviations.

**TABLE 2:** Derivation of DNA content per tissue mass from mean DNA concentration for untreated tissue, Protocol A, and Protocol B.

Sample type	Biopsy dry weight (mg)	DNA concentration (ng/µL)	DNA content (ng/mg)	Mean (ng/mg)	SD
Untreated	4.00	21.73	163.00	190.92	27.92
	3.45	21.96	190.92		
	2.90	21.15	218.84		
Protocol A	3.80	1.06	8.35	9.25	0.90
	3.50	1.08	9.25		
	3.00	1.02	10.15		
Protocol B	3.50	0.028	0.24	4.95	4.71
	3.80	0.63	4.95		
	3.40	1.09	9.66		

**TABLE 3:** Derivation of DNA content per tissue mass from mean DNA concentration for untreated tissue and Protocol C.

Sample type	Biopsy dry weight (mg)	DNA concentration (ng/µL)	DNA content (ng/mg)	Mean (ng/mg)	SD
Untreated	3.20	18.34	171.98	196.69	24.71
	3.41	22.36	196.69		
	3.12	23.03	221.40		
Protocol C	3.81	1.22	9.58	11.53	1.95
	3.42	1.31	11.53		
	3.43	1.54	13.48		

### 3.2 IHC staining and quantification of ECM proteins

IHC staining was performed to localize and quantify key basement membrane and structural ECM proteins, including collagen IV (Col IV), elastin, and laminin, in both untreated skin tissue and ADM scaffolds prepared using the three protocols (A-C). All three ECM proteins were retained following decellularization, although retention levels varied between protocols.

**FIGURE 2:**
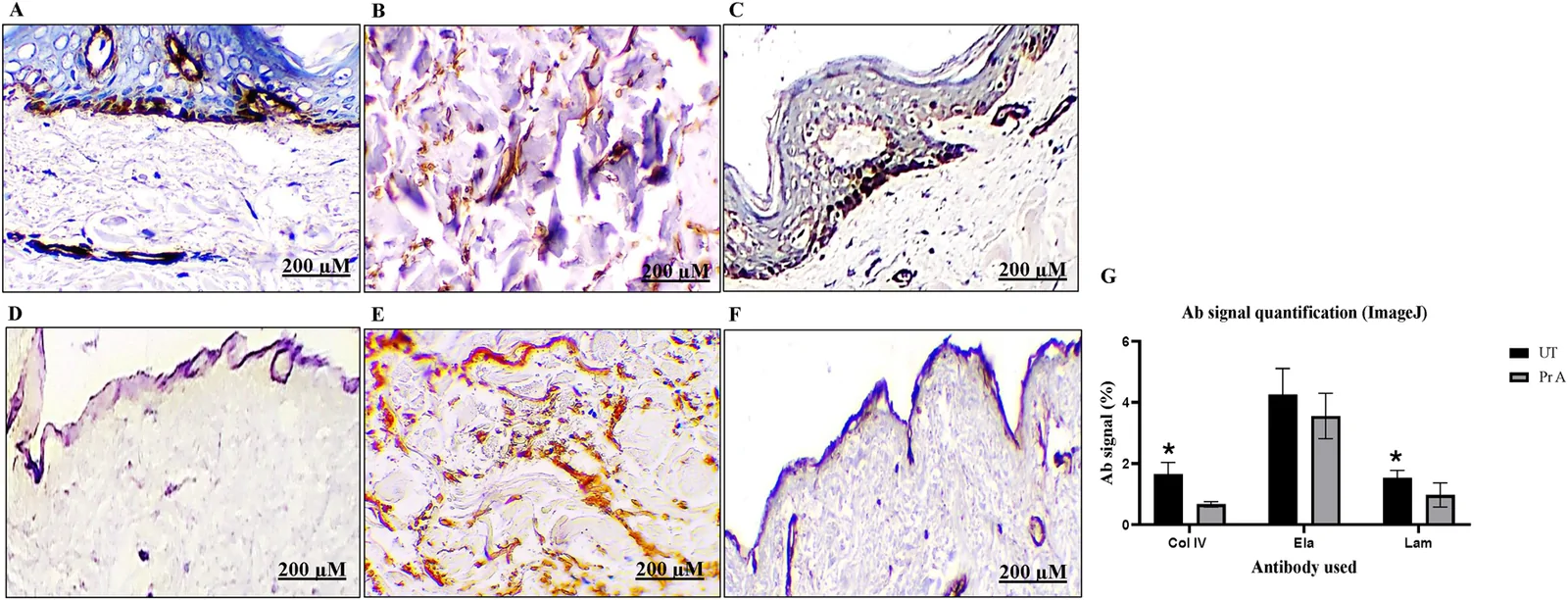
Immunohistochemical visualization of collagen IV, elastin, and laminin in untreated tissue and tissue decellularized using Protocol A. **(A–C)** show collagen IV, elastin, and laminin, respectively, in untreated tissue. **(D–F)** show collagen IV, elastin, and laminin, respectively, in Protocol A scaffolds. **(G)** shows ImageJ quantification of the staining for these proteins. Each protocol was performed three times (n = 3); data are presented as mean ± SD. *p < 0.05.

**FIGURE 3:**
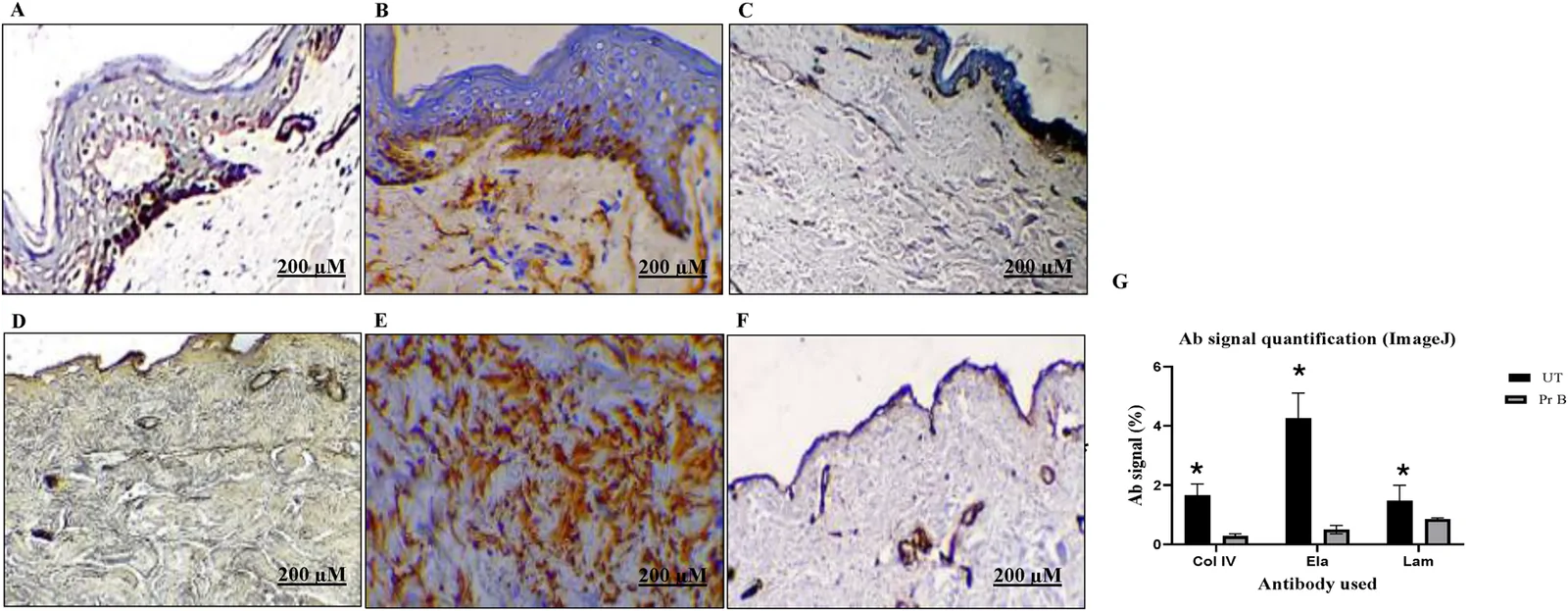
Immunohistochemical visualization of collagen IV, elastin, and laminin in untreated tissue and tissue decellularized using Protocol B. **(A–C)** show collagen IV, elastin, and laminin, respectively, in untreated tissue. **(D–F)** show collagen IV, elastin, and laminin, respectively, in Protocol B scaffolds. **(G)** shows ImageJ quantification of staining for these proteins. Experiments were performed three times (n = 3); data are presented as mean ± SD. *p < 0.05.

**FIGURE 4:**
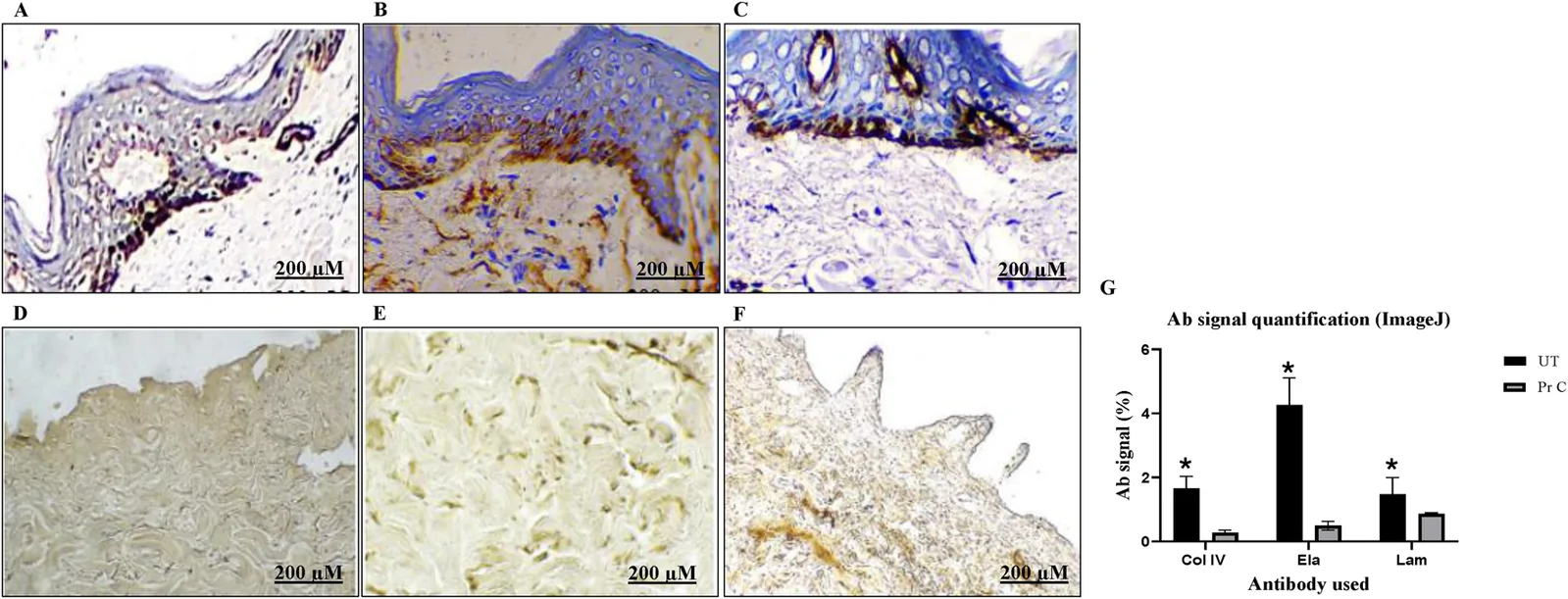
Immunohistochemical visualization of proteins of interest (collagen IV, elastin, and Laminin) that play a vital role in wound healing. **(A–C)** represent antibody detection from normal untreated tissue samples and from **(A–C)** is collagen IV, elastin, and Laminin respectively. **(D–F)** represent antibody detections from Protocol C methods and from **(D–F)** is collagen IV, elastin, and Laminin respectively. **(G)** shows the quantification (%) of these proteins using ImageJ software. Each protocol was performed three times (n=3), data are presented as mean ± SD. * p<0.05.

For Collagen IV, IHC staining was conducted on both untreated and ADM sections using an antibody concentration of 1:400. Positive signals were observed in both conditions, confirming the presence of Col IV. Collagen IV was observed to be localized in the ECM basement membrane zone, demarcating epithelial and endothelial interfaces. The results for Collagen IV are illustrated in Figures 2–4A,D,G,L and 4A,D. Quantitative ImageJ analysis demonstrated protocol-dependent differences in ECM protein retention.

Elastin staining was performed at an antibody concentration of 1:100, yielding a strong positive signal in both untreated and ADM sections. Elastin was distributed throughout the dermis.

Laminin staining was conducted at a concentration of 1:400 with antigen retrieval. A strong positive signal was observed in both untreated and ADM sections, with high intensity around blood vessels and beneath the basement membrane (Figures 2C,F; 3C,F; and 4C,F). Laminin staining, at the same concentration (1:400) without antigen retrieval also demonstrated a positive signal in both untreated and ADM sections, with high intensity in similar locations (Figures 2–4C,F).

These findings indicate that collagen IV, elastin, and laminin were retained following decellularization, supporting preservation of critical ECM components essential for scaffold biofunctionality. Notably, laminin and elastin appeared to exhibit greater preservation relative to collagen IV, which may be particularly relevant for maintaining basement membrane integrity and biomechanical function.

**FIGURE 5:**
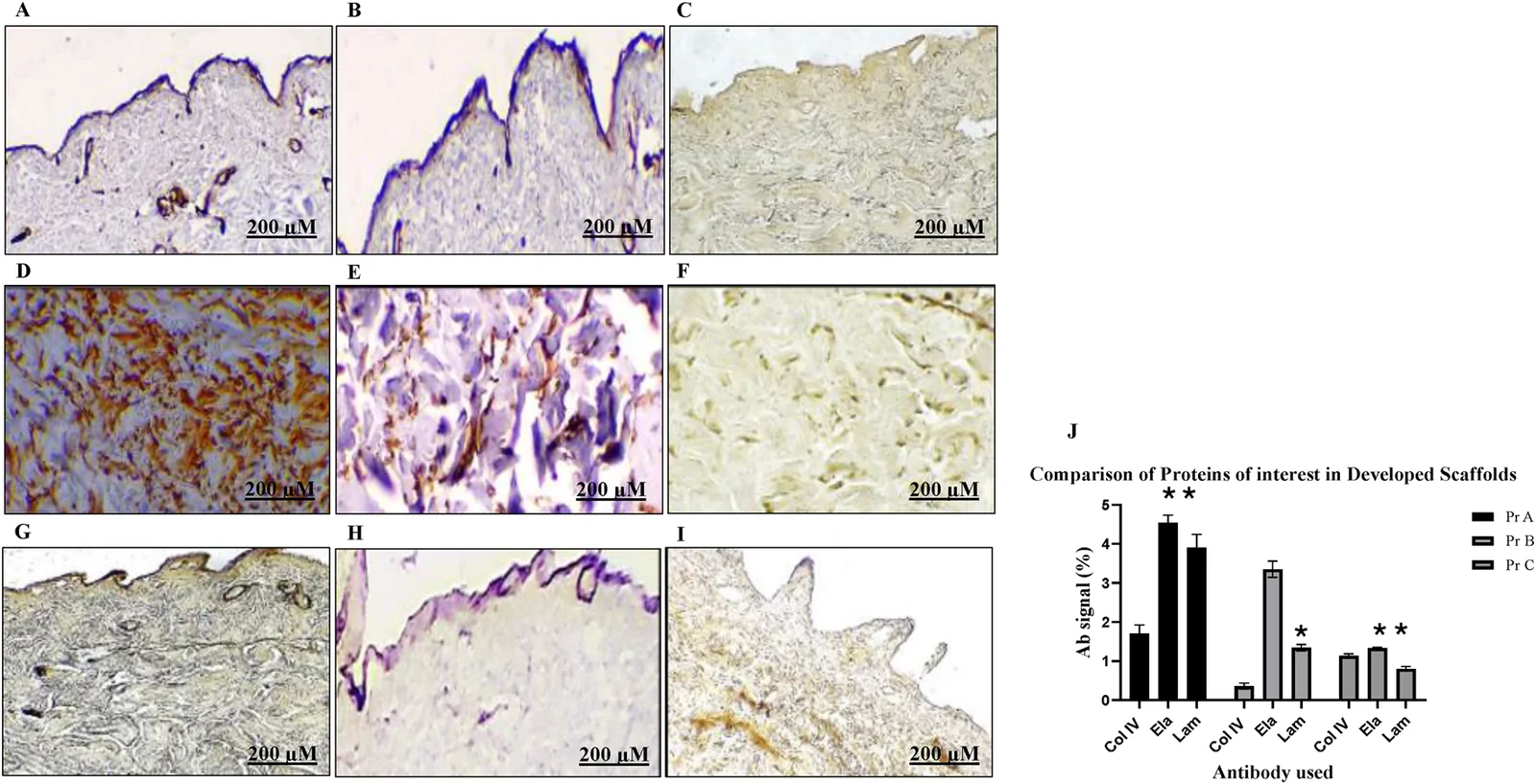
Immunohistochemical comparison of extracellular matrix proteins of interest (collagen IV, elastin, and laminin) among Protocols A–C. **(A–C)** show antibody staining for collagen IV in tissues processed using Protocols A, B, and C, respectively. **(D–F)** show antibody staining for elastin in tissues processed using Protocols A, B, and C, respectively. **(G–I)** show antibody staining for laminin in tissues processed using Protocols A, B, and C, respectively. **(J)** Quantitative analysis (%) of collagen IV, elastin, and laminin expression was performed using ImageJ software, comparing protein preservation among the three decellularization protocols. Each protocol was performed in triplicate (n = 3), and data are presented as mean ± standard deviation (SD). *p < 0.05 and **p < 0.01. Abbreviations: Ab, antibody; Col IV, collagen IV; Ela, elastin; Lam, laminin; Pr A, Protocol A; Pr B; Protocol B; Pr C, Protocol C.

Comparative analysis revealed protocol-dependent differences in ECM protein retention, with ADM sections demonstrating varying degrees of protein loss following chemical treatment. Among the protocols, Protocol A retained the highest levels of all three proteins, followed by Protocol B, while Protocol C demonstrated the lowest protein retention (p < 0.05, one-way ANOVA with Tukey’s *post hoc* test). A comparative summary of the IHC results from the three protocols is presented in Figures 5A–I, with ImageJ quantification results shown in Figure 5J.

Since collagen I and III did not yield optimal signal specificity during antibody optimization, histochemical staining approaches were employed to assess overall collagen and elastic fiber architecture. Masson’s Trichrome and Verhoeff’s Van Gieson (VVG) staining, were performed on untreated and ADM sections to evaluate tissue structure and ECM composition.

**FIGURE 6:**
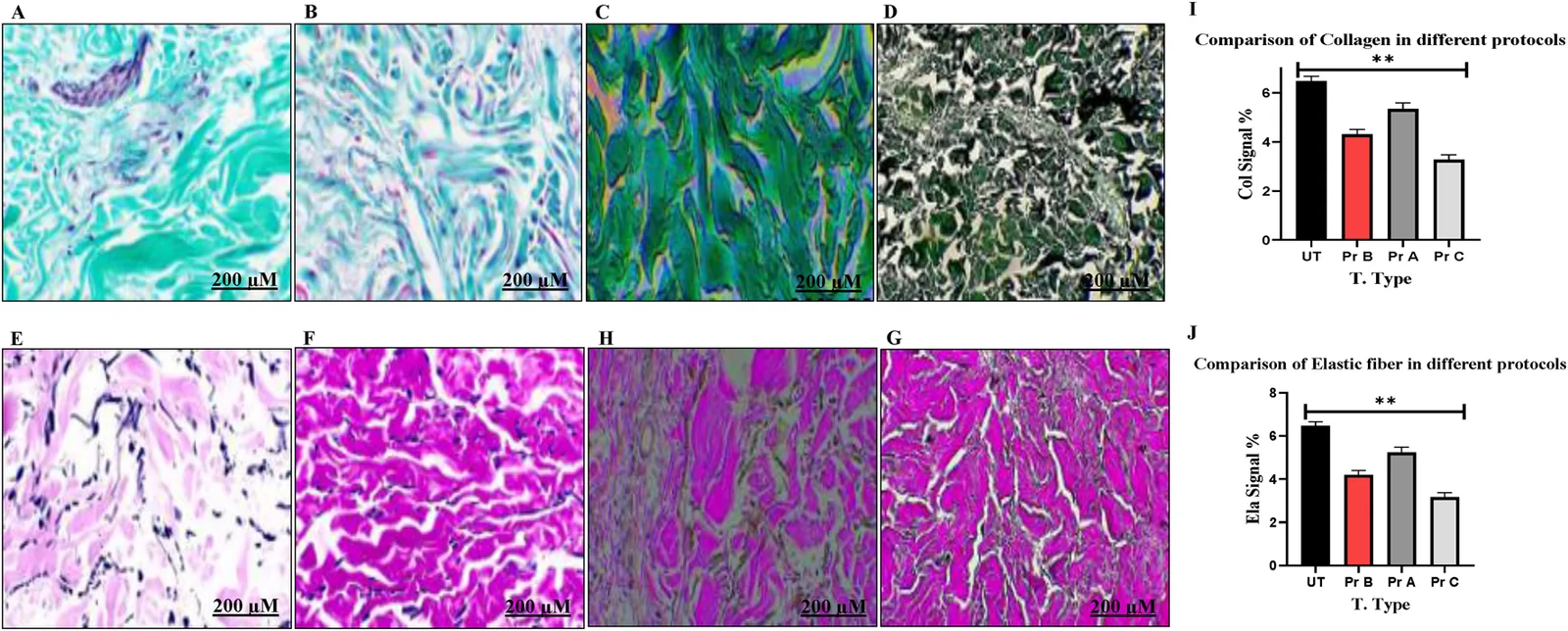
Comparison of collagen and elastic fiber preservation in tissues processed using Protocols A–C, assessed by trichrome and elastic fiber staining. **(A,E)** represent untreated tissue (UT) controls. **(B,F)** represent tissues processed using Protocol B. **(C,G)** represent tissues processed using Protocol A. **(D,H)** represent tissues processed using Protocol C. **(I,J)** Quantitative analysis (%) of collagen and elastic fiber content was performed using ImageJ software. Each protocol was performed in triplicate (n = 3), and data are presented as mean ± standard deviation (SD). **p < 0.01. Abbreviations: Col, collagen; UT, untreated tissue; Pr A, Protocol A; Pr B, Protocol B; Pr C, Protocol C; T, tissue.

Masson’s Trichrome staining was used to selectively highlight collagen within tissue sections. In untreated samples, the staining revealed a clear distinction between the epidermis and dermis, with well-defined collagen staining indicative of a healthy, intact ECM. ADM sections from the three protocols displayed varying degrees of ECM preservation, characterized by different collagen network levels. Protocol A demonstrated the highest collagen retention, followed by Protocol B, while Protocol C exhibited disrupted collagen architecture (Figures 6A–D, with Figure 6I showing collagen quantification).

Verhoeff’s Van Gieson staining was used to assess pathological changes in elastic fibers, including thinning and fragmentation. In both untreated and ADM sections, elastin fibers were identified as black-stained structures distributed throughout the dermis (Figures 6E–H, with Figure 6J illustrating elastic fiber quantification). Protocol A showed conservation of elastic fibers, Protocol B demonstrated lower expression than Protocol A, and Protocol C indicated the lowest levels of elastic fibers compared to the other two protocols.

### 3.3 Raman spectroscopy analysis

Raman spectroscopy was used to evaluate preservation of ECM-associated molecular signatures following decellularization. Raman spectroscopy was performed on ADM samples to evaluate preservation of characteristic ECM-associated molecular signatures following decellularization. Post-treatment, the slides were analyzed using Raman spectroscopy on a Tescan MIRA3 RISE SEM platform and identified characteristic Raman spectral signatures associated with collagen, elastin, and laminin in ADM samples.

**FIGURE 7:**
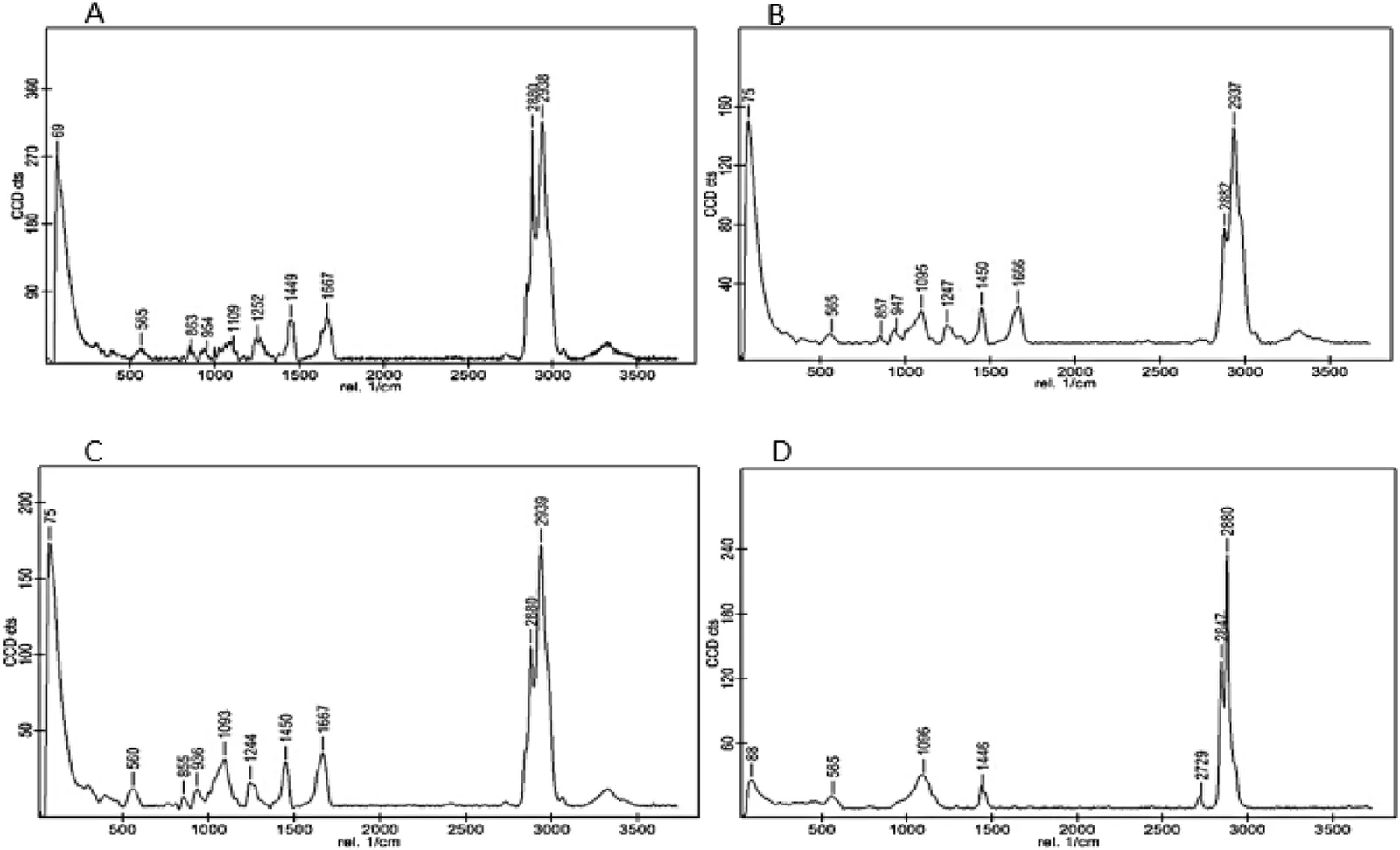
Raman spectroscopy analysis of native and decellularized tissue samples. **(A)** Raman spectrum of untreated tissue. **(B)** Raman spectrum of tissue processed using Pr A. **(C)** Raman spectrum of tissue processed using Pr B. **(D)** Raman spectrum of tissue processed using Pr C. Characteristic peaks correspond to protein-associated chemical groups present in the tissue samples, providing information on biochemical composition and structural preservation following decellularization. Abbreviation. Pr, protocol.

Protocols A and B demonstrated clear preservation of Raman peaks corresponding to the targeted ECM proteins (Figures 7B,C. Panel A is untreated tissue; panels B and C are Protocols A and B), while Protocol C exhibited reduced or attenuated Raman signals, suggesting partial loss or alteration of specific ECM components (Figure 7D).

### 3.4 Morphological (SEM) analysis

**FIGURE 8:**
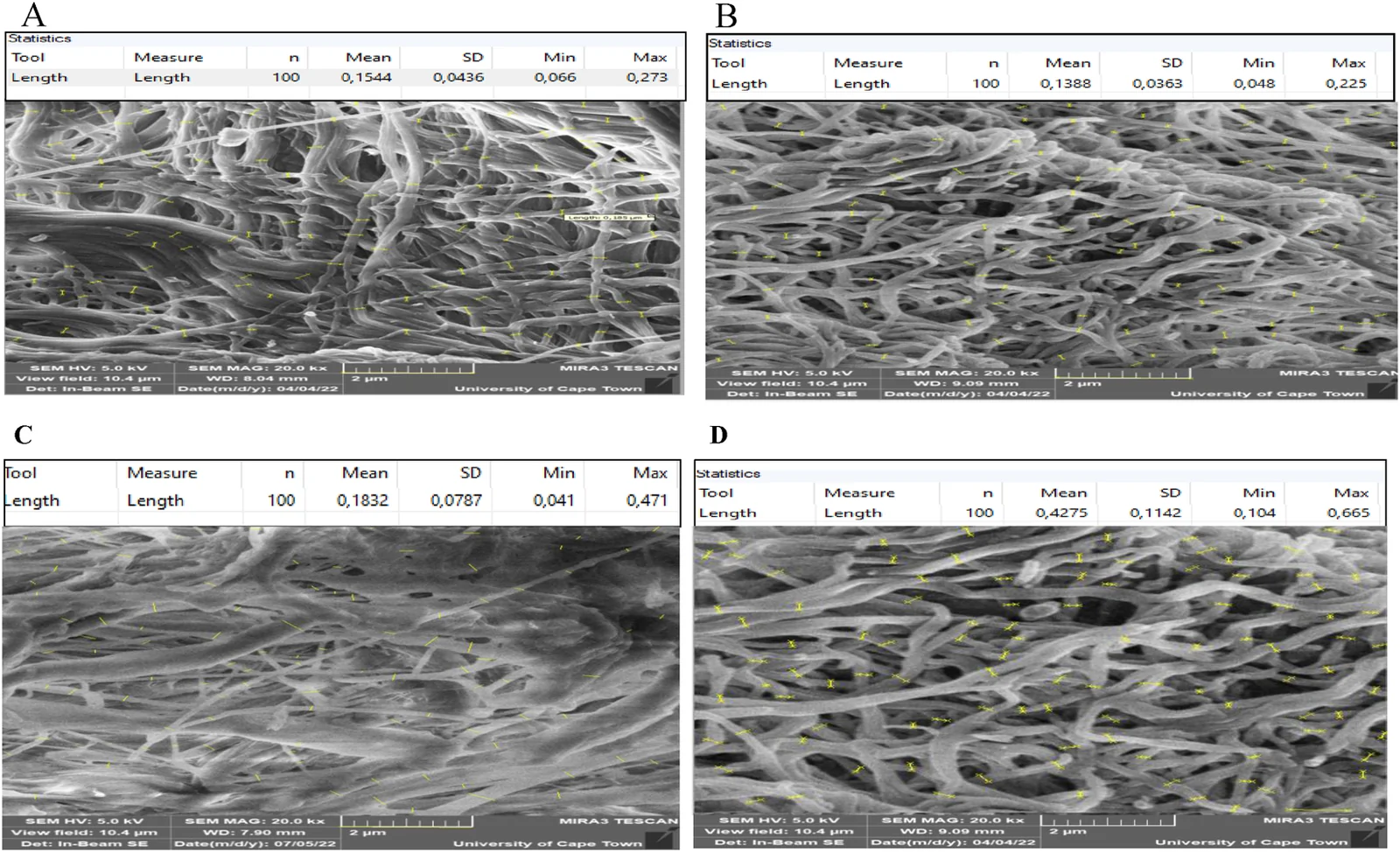
Representative scanning electron microscopy images showing tissue surface topography and fibre architecture. Fibre dimensions were measured using Digimizer software. **(A)** Untreated tissue, **(B)** Protocol A, **(C)** Protocol B, **(D)** Protocol C.

Given the observed preservation of ECM-associated molecular signatures, scaffold ultrastructure was next evaluated using SEM analysis. Morphology and topography are biological properties of ADM scaffolds that influence the ability to support cell attachment, proliferation, and tissue remodelling. High-resolution SEM images validated successful decellularization, conservation of surface integrity, and apparent increase in microstructural openness in ADM samples in all protocols (A–C) (Figures 8A–D). Collagen fiber width was analyzed using Digimizer software by measuring the diameter of 100 randomly selected fibers per image. Collagen fiber width analysis revealed that chemical treatment significantly influenced fiber dimensions (p < 0.01, one-way ANOVA). Protocol A exhibited the smallest collagen fiber width (0.1388 ± 0.0363 µm), closely resembling the untreated sample’s collagen width, followed by Protocol B (0.18 ± 0.0787 µm). Protocol C showed the largest fiber width (0.4275 ± 0.1142 µm), suggesting that increased chemical exposure may disrupt collagen ultrastructure, potentially affecting mechanical integrity and cell–matrix interactions.

### 3.5 Mechanical properties evaluation using a universal tensile tester

**FIGURE 9:**
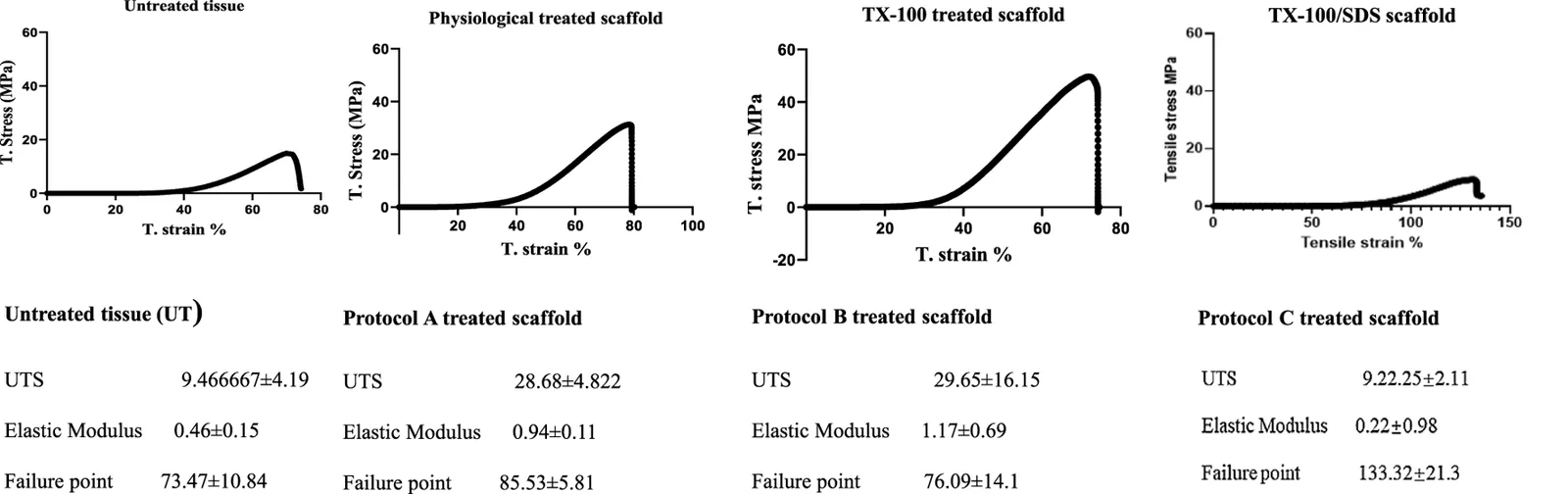
The universal tensile tester was used to evaluate the maximum stress of the samples before and after tissue treatment. Results are presented as ultimate tensile strength (UTS), elastic modulus, and failure point.

Mechanical testing revealed distinct differences in tensile behavior between untreated skin and ADM scaffold samples. The comparison of mechanical properties across all protocols (untreated and ADM samples) revealed key differences in ultimate tensile strength (UTS) and elastic modulus. The UTS of untreated samples was significantly lower compared to Protocols A and B (p < 0.05, one-way ANOVA), whereas Protocol C exhibited lower tensile strength than untreated tissue. Elastic modulus measurements indicated that untreated samples and Protocol C had lower stiffness and higher tensile strain, whereas Protocols A and B exhibited increased elastic modulus values (Figure 9). These findings suggest that low-detergent decellularization preserves or enhances mechanical strength and performance, whereas Protocol C demonstrated reduced mechanical performance.

### 3.6 ADM functionality and cell proliferation

The metabolic activity and proliferation of fibroblasts seeded on the ADM scaffolds were assessed over a 21-day period using the PrestoBlue assay. Absorbance values were recorded on days 7, 14, and 21. Measurements obtained on days 7, 14, and 21 demonstrated a progressive and statistically significant increase in metabolic activity and fibroblast proliferation over time (p < 0.01, one-way ANOVA).

By day 14, a notable increase in absorbance indicated strong cell adhesion and proliferation, and by day 21, fibroblasts on the ADM samples exhibited a marked increase in absorbance, suggesting substantial scaffold repopulation. Compared to the control group, the ADM samples provided a superior microenvironment for fibroblast proliferation, with Protocols A and B demonstrating enhanced fibroblast proliferation. However, Protocol C demonstrated reduced proliferative support.

These findings were further validated by the WST-1 assay, which produced similar outcomes, confirming that Protocol A demonstrated greater functional performance than Protocol B, and Protocol B outperformed Protocol C.

**FIGURE 10:**
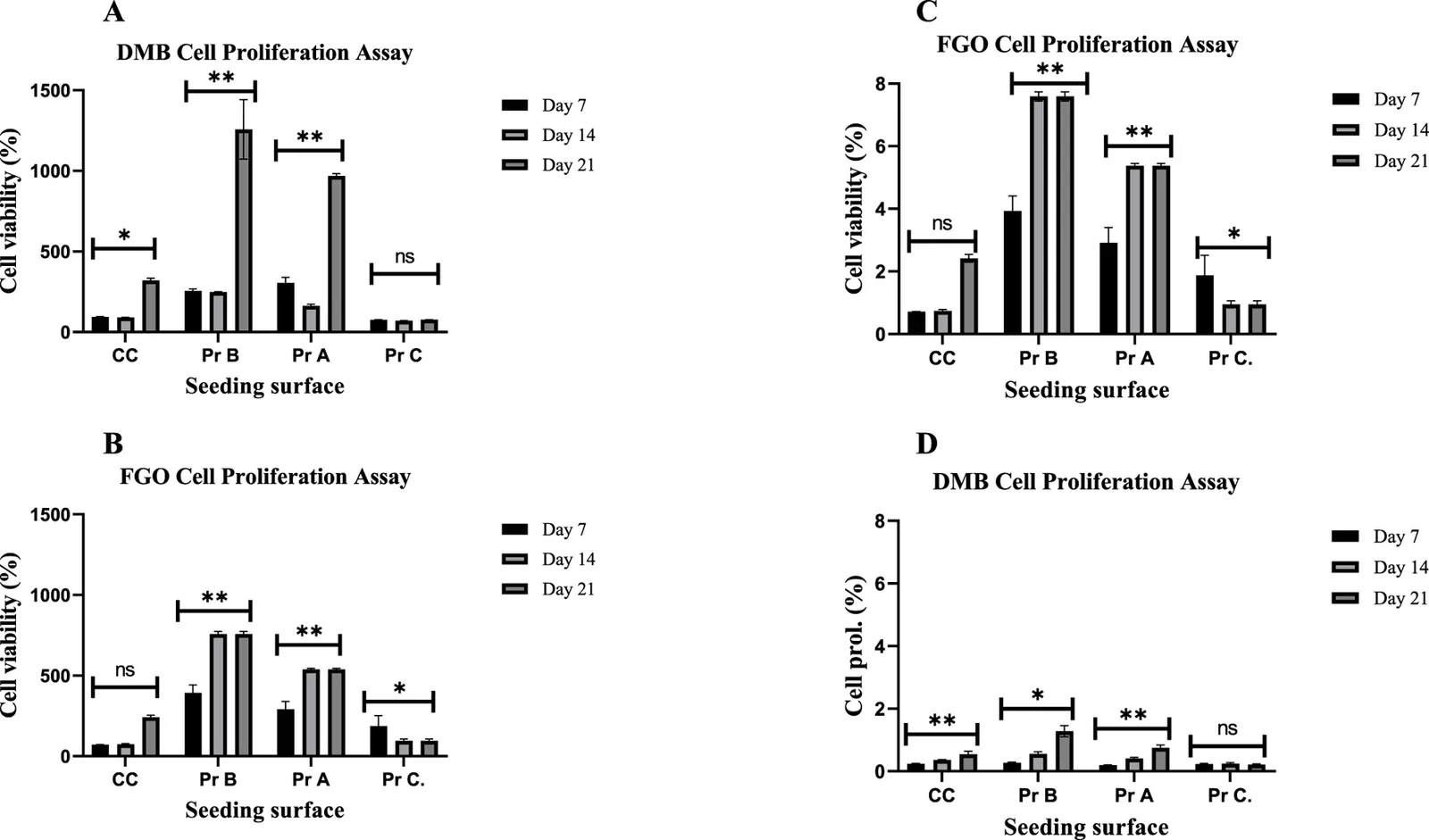
Cell viability and proliferation of DMB and FGO fibroblasts cultured on scaffolds generated using Protocols A–C. **(A)** DMB and **(B)** FGO fibroblast viability measured using PrestoBlue. **(C)** FGO and **(D)** DMB fibroblast proliferation measured using WST-1.

Overall, Protocol A demonstrated the highest functional performance, followed by Protocol B, while Protocol C consistently underperformed. These results are summarized in Figure 10.

### 3.7 *Ex vivo* model: scaffold integration capacity and cellular interaction

**FIGURE 11:**
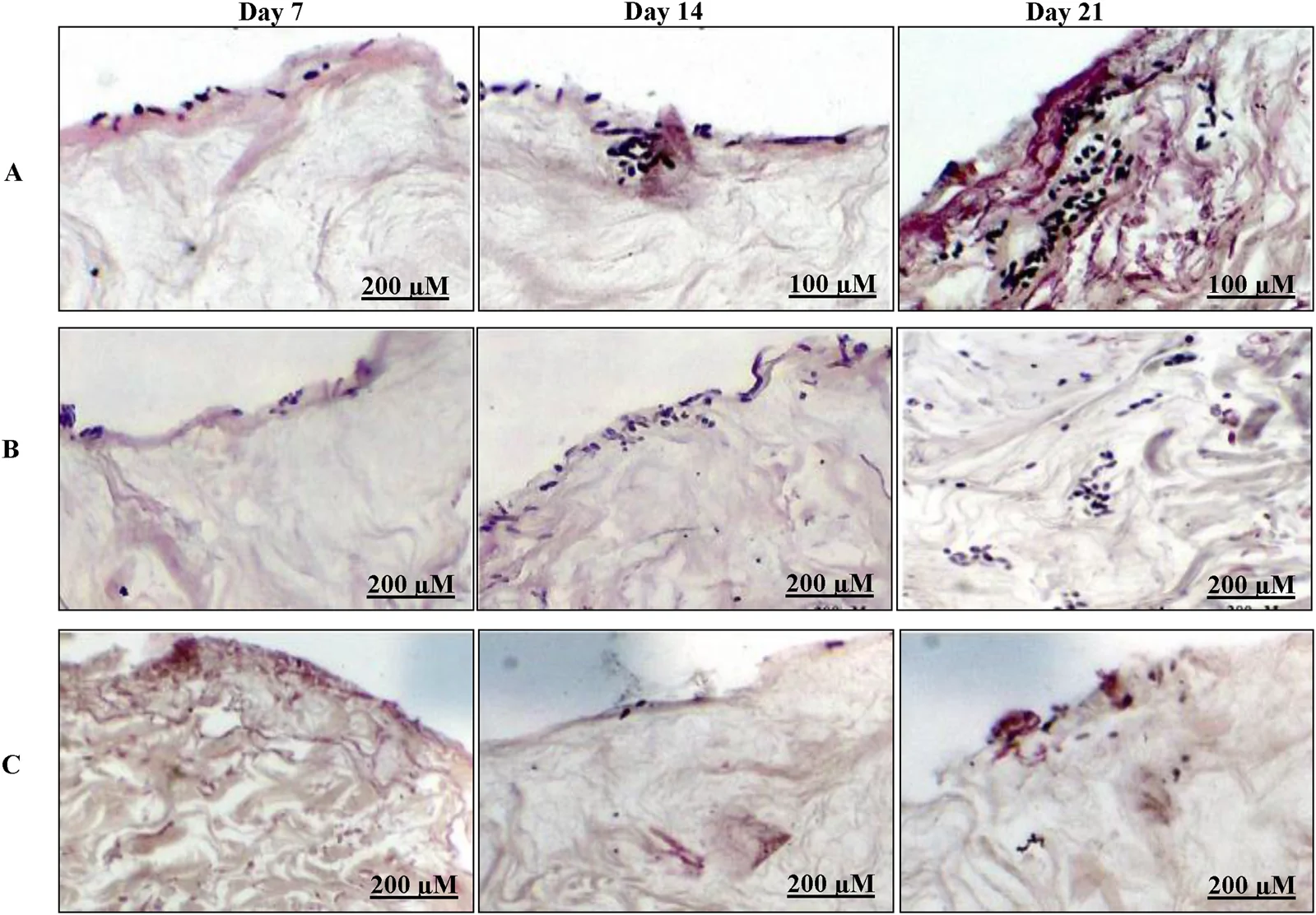
Ex vivo analysis showing the distribution of cell propagation from fresh tissue within the Protocol A **(A)**, Protocol B **(B)**, and Protocol C **(C)** ADM.

Protocol B is presented first as a representative example of *ex vivo* integration kinetics prior to the full cross-protocol comparison in Section 3.8. The *ex vivo* model using Protocol B ADM scaffolds integrated with fresh human skin tissue, demonstrated successful fibroblast interaction with the ADM over a 21-day incubation period. Histological analysis has shown that by day 7, fibroblasts had begun to infiltrate the scaffold, with 286 fibroblasts observed. By day 14, fibroblast penetration increased significantly, reaching 564 fibroblasts. At day 21, the scaffold exhibited extensive cellular repopulation with 749 fibroblasts distributed in the dermis, indicating successful integration and proliferation of the fibroblasts within the ADM scaffold. Figure 11 shows *ex vivo* analysis investigating the distribution of cells from fresh tissue within an ADM scaffold over three time points: 7, 14, and 21 days. These findings support the capacity of the ADM scaffold to facilitate cellular integration *ex vivo* and support its suitability for regenerative and reconstructive applications.

### 3.8 *Ex Vivo* model for assessing acellular scaffold integration capacity through cell propagation from fresh human tissue

#### 3.8.1 H&E staining of tissue sections at 7, 14, and 21 days

**FIGURE 12:**
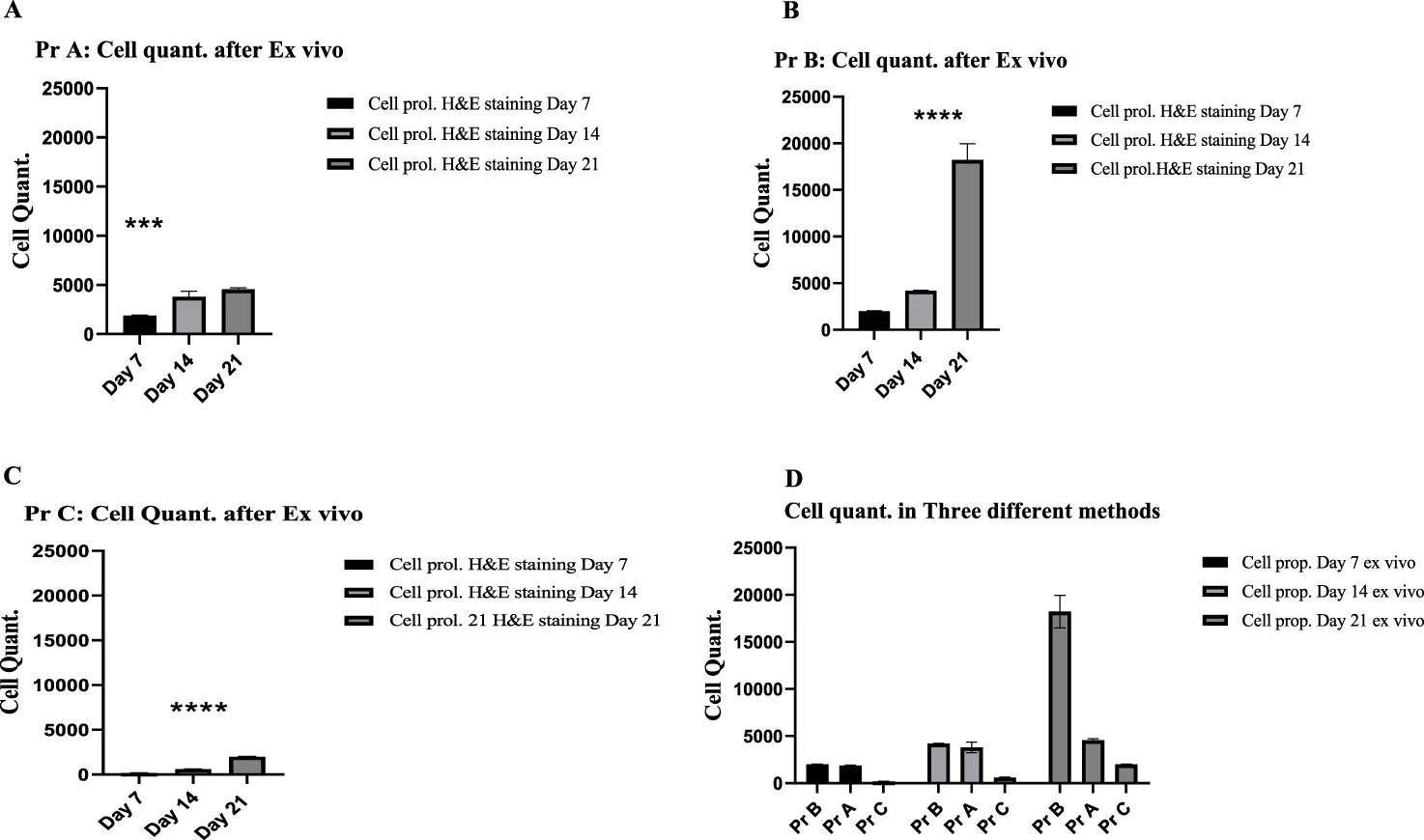
Quantitative analysis of cell proliferation within the scaffolds. (A–C) ImageJ-based cell quantification of scaffold sections, with data presented using GraphPad Prism. (D) Comparison of cell proliferation among the different scaffold types. Data are presented as mean ± standard deviation (SD). ***p < 0.001 and ****p < 0.0001. Abbreviations: Quant., quantification; H&E, haematoxylin and eosin; Pr, protocol.

The *ex vivo* model for Protocols A, B, and C demonstrated successful cell propagation within the ADMs over 7, 14, and 21 days (Figure 11). Progressive cellular infiltration from the surrounding native tissue into the ADM scaffold was evident across all protocols, indicating scaffold permissiveness to host-derived cell migration. Protocol B showed the most robust growth, with cell numbers increasing from day 7 to approximately 20,000 by day 21 (Figure 12B). Statistical analysis confirmed a significant difference among group means (p = 0.0001) with an *R* ^2^ value of 0.9872, indicating a strong model fit. This high coefficient of determination reflects a consistent and reproducible proliferation trend over time.

Protocol A also supported substantial cell proliferation, reaching approximately 5,000 cells by day 21 (Figure 12A), with statistical significance (p = 0.0002) and a strong correlation (*R* ^2^ = 0.9427). Although less pronounced than Protocol B, Protocol A maintained sustained cellular ingress and expansion throughout the culture period. Protocol C exhibited measurable but limited cell growth, with numbers reaching just under 2,000 by day 21 (Figures 11C, 12C), and statistical analysis indicated a significant difference among groups (p = 0.0001, *R* ^2^ = 0.9981). This suggests that more aggressive chemical decellularization may impair scaffold-mediated cell propagation.

Overall, Protocol B (0.1% Triton X-100) consistently demonstrated the most robust cell migration and proliferation in this *ex vivo* model, reaching approximately 20,000 cells per 4-mm punch by day 21, whereas Protocol A supported approximately 5,000 cells and Protocol C fewer than 2,000 cells (Figures 12A–D). These findings indicate that reduced-detergent decellularization may provide a favorable balance between cellular clearance and preservation of the biological cues required for tissue repopulation.

#### 3.8.2 Immunohistochemistry and quantification of collagen IV, elastin, and laminin in the ex vivo model at 7, 14, and 21 days

**FIGURE 13:**
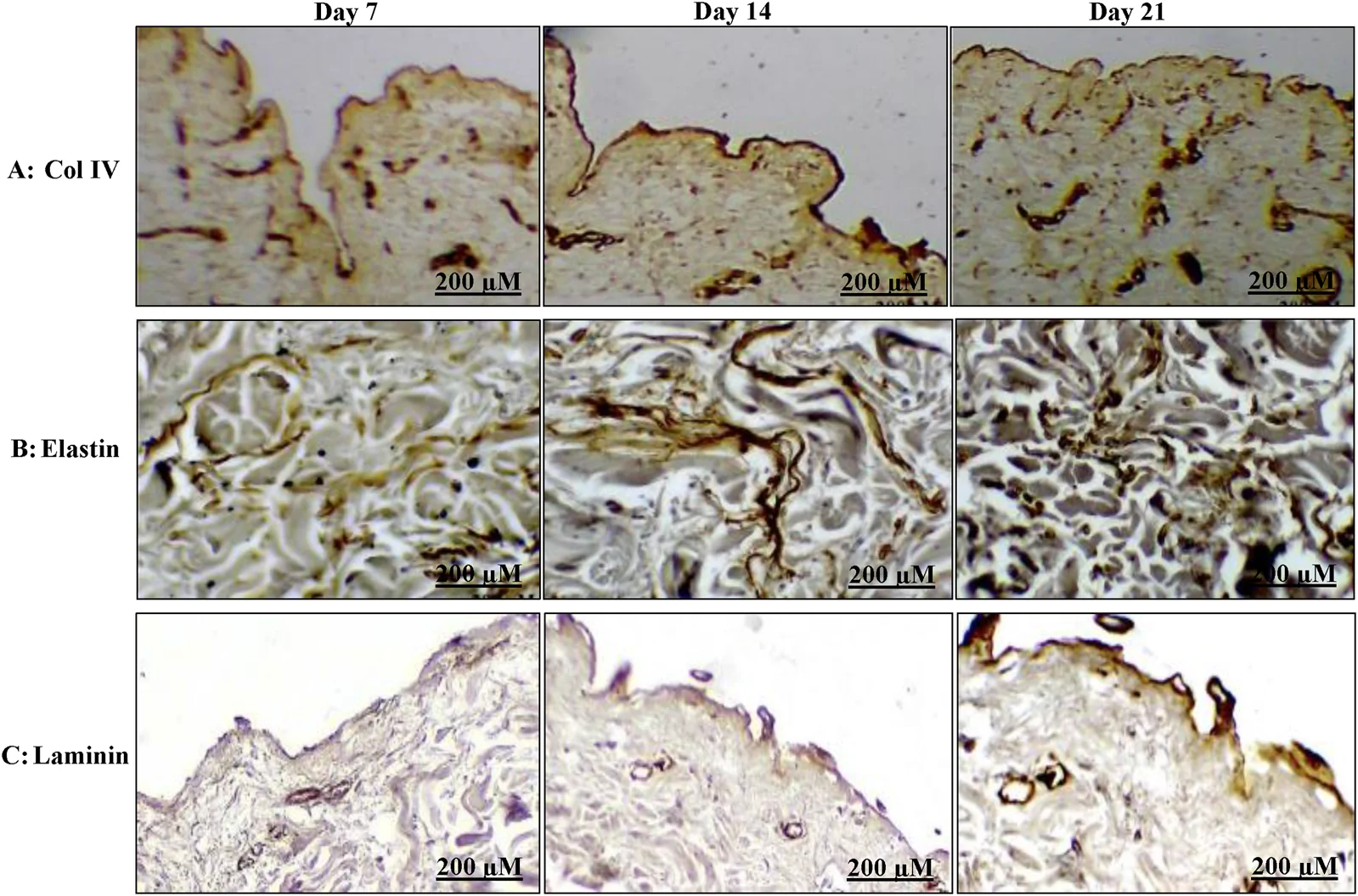
Immunohistochemical expression of collagen IV, elastin, and laminin in Protocol A scaffolds following ex vivo incubation with fresh human tissue for 7, 14, and 21 days. Rows A–C show collagen IV, elastin, and laminin, respectively; columns from left to right show days 7, 14, and 21. Abbreviations: Col IV, collagen IV; Pr A, Protocol A.

**FIGURE 14:**
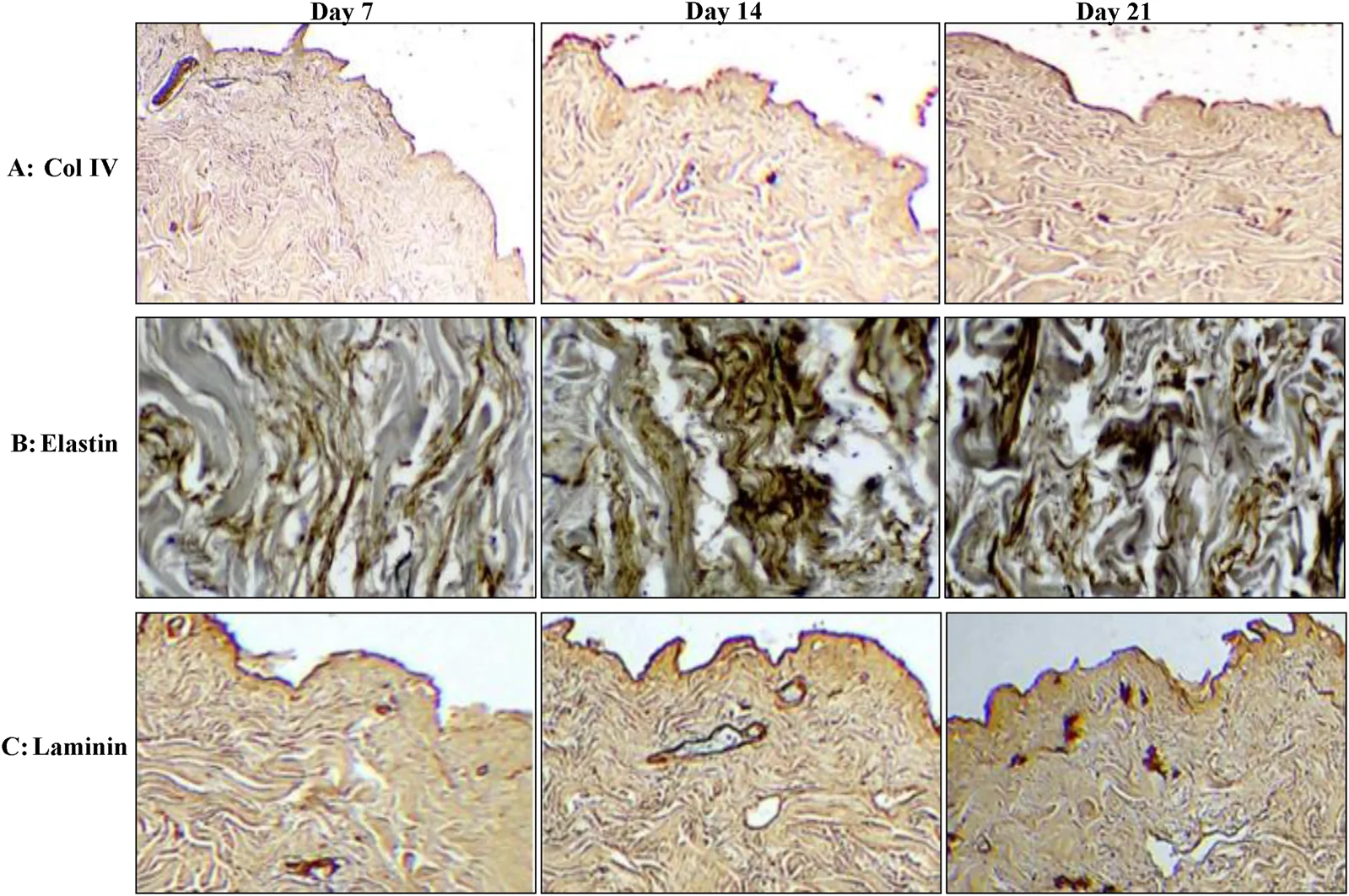
Immunohistochemical expression of collagen IV, elastin, and laminin in Protocol B scaffolds following ex vivo incubation with fresh human tissue for 7, 14, and 21 days. Rows A–C show collagen IV, elastin, and laminin, respectively; columns from left to right show days 7, 14, and 21. Abbreviations: Col IV, collagen IV; Pr B, Protocol B.

**FIGURE 15:**
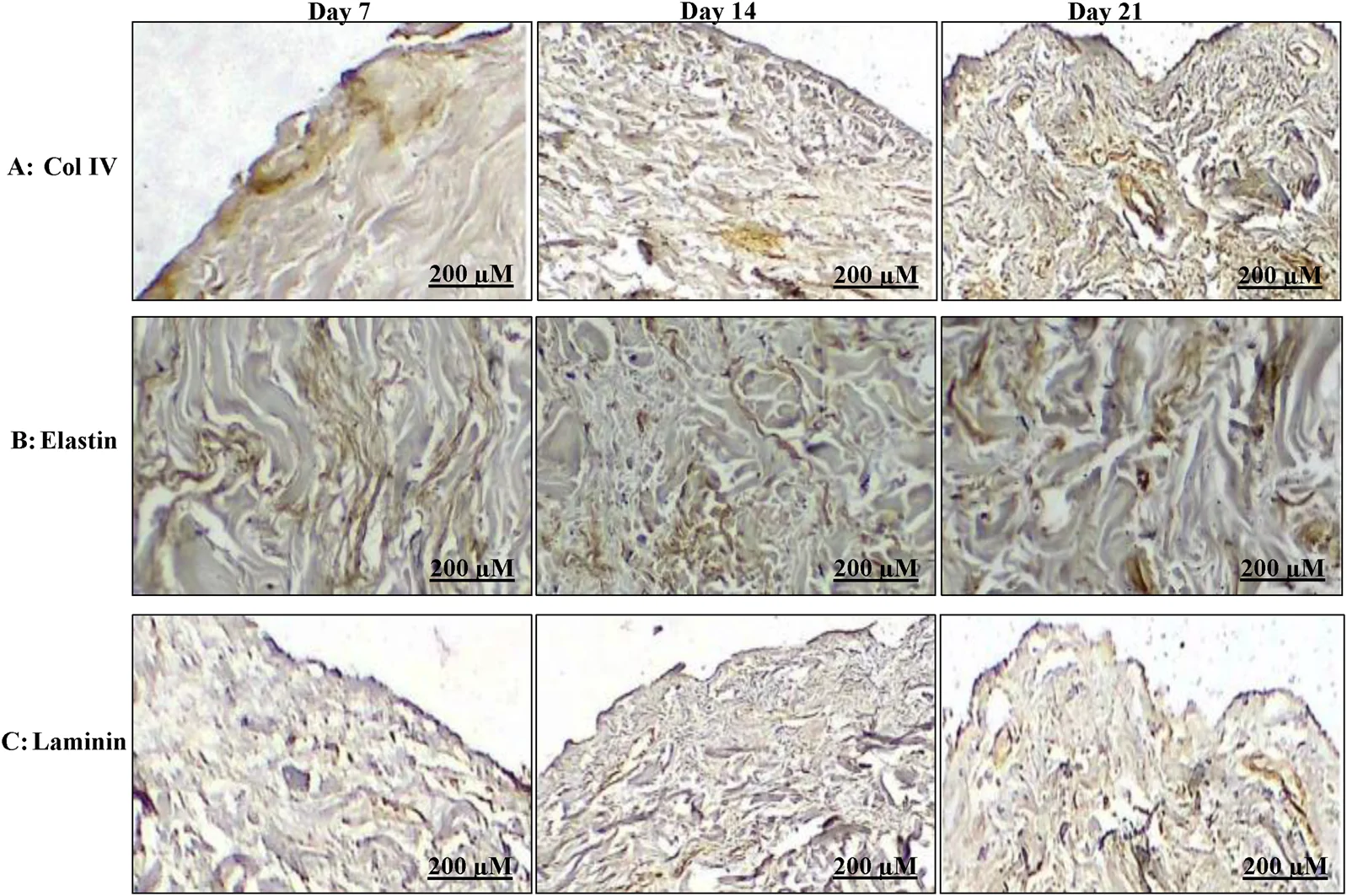
Immunohistochemical expression of collagen IV, elastin, and laminin in Protocol C scaffolds following ex vivo incubation with fresh human tissue for 7, 14, and 21 days. Rows A–C show collagen IV, elastin, and laminin, respectively; columns from left to right show days 7, 14, and 21. Abbreviations: Col IV, collagen IV; Pr C, Protocol C.

**FIGURE 16:**
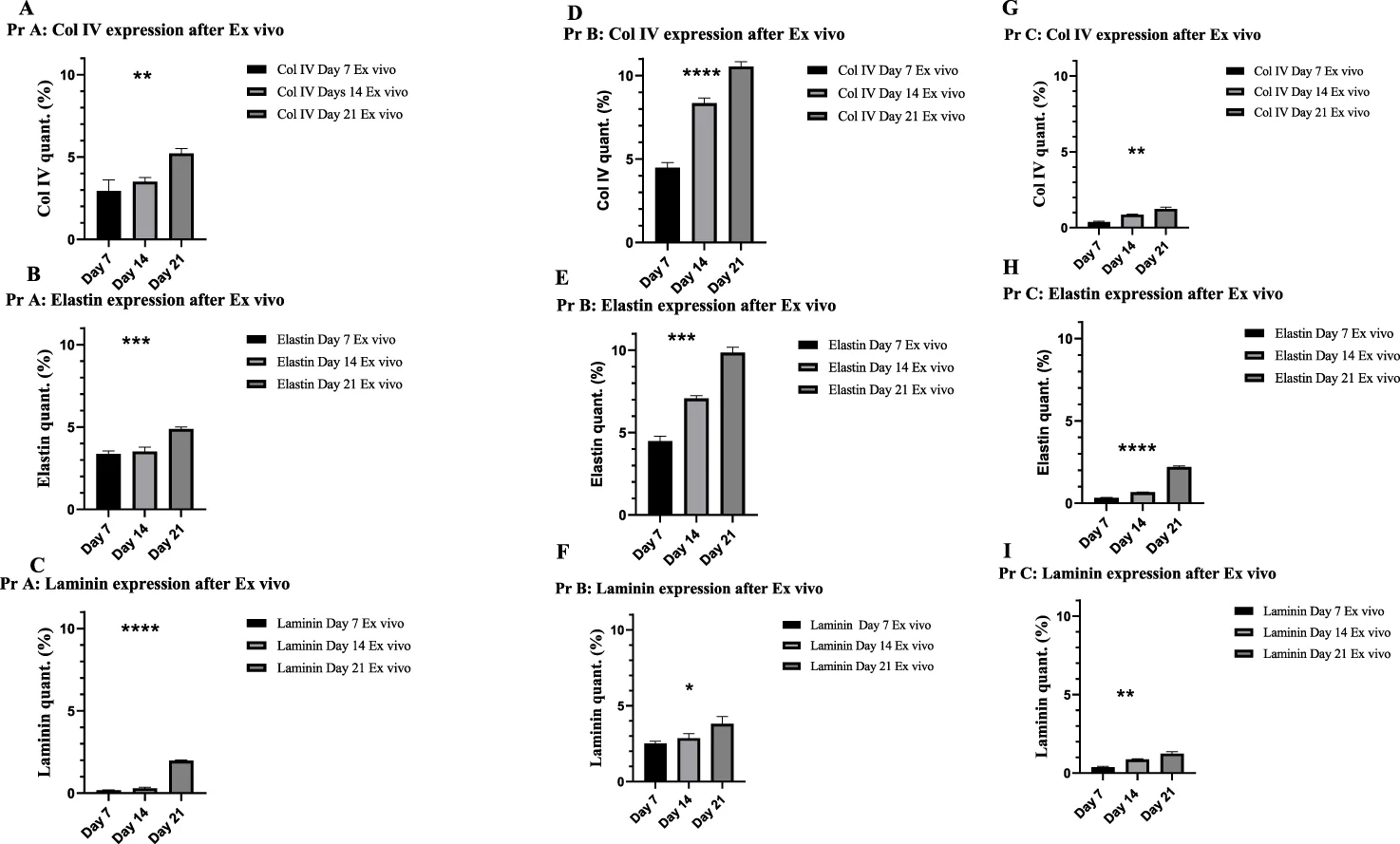
Comparative analysis of collagen IV, elastin, and laminin expression in the scaffolds. (**A–C**, **D–F**, and **G–I** for Protocols A, B, and C, respectively) ImageJ-based quantification of protein expression, analyzed and presented using GraphPad Prism. Each experiment was performed in triplicate (n = 3), and data are presented as mean ± standard deviation (SD). *p < 0.05, **p < 0.01, ***p < 0.001, and ****p < 0.0001. Abbreviations: Col IV, collagen IV; Quant., quantification.

IHC staining was performed to localize and quantify three ECM proteins—collagen IV (Col IV), elastin, and laminin—in bio-scaffolds incubated with fresh human skin for 7, 14, and 21 days under Protocols A–C. This approach enabled assessment of both scaffold preservation and *de novo* ECM deposition during *ex vivo* remodeling. This analysis evaluated changes in protein expression over time. Across all protocols, ECM protein levels increased from day 7 to day 21 (Figures 13–16), with Protocol B showing the greatest increase, followed by Protocol A, while Protocol C exhibited the smallest increase (Figures 15A–C; Figures 16G–I). The progressive increase in ECM proteins suggests active matrix remodeling driven by infiltrating host cells.

**FIGURE 17:**
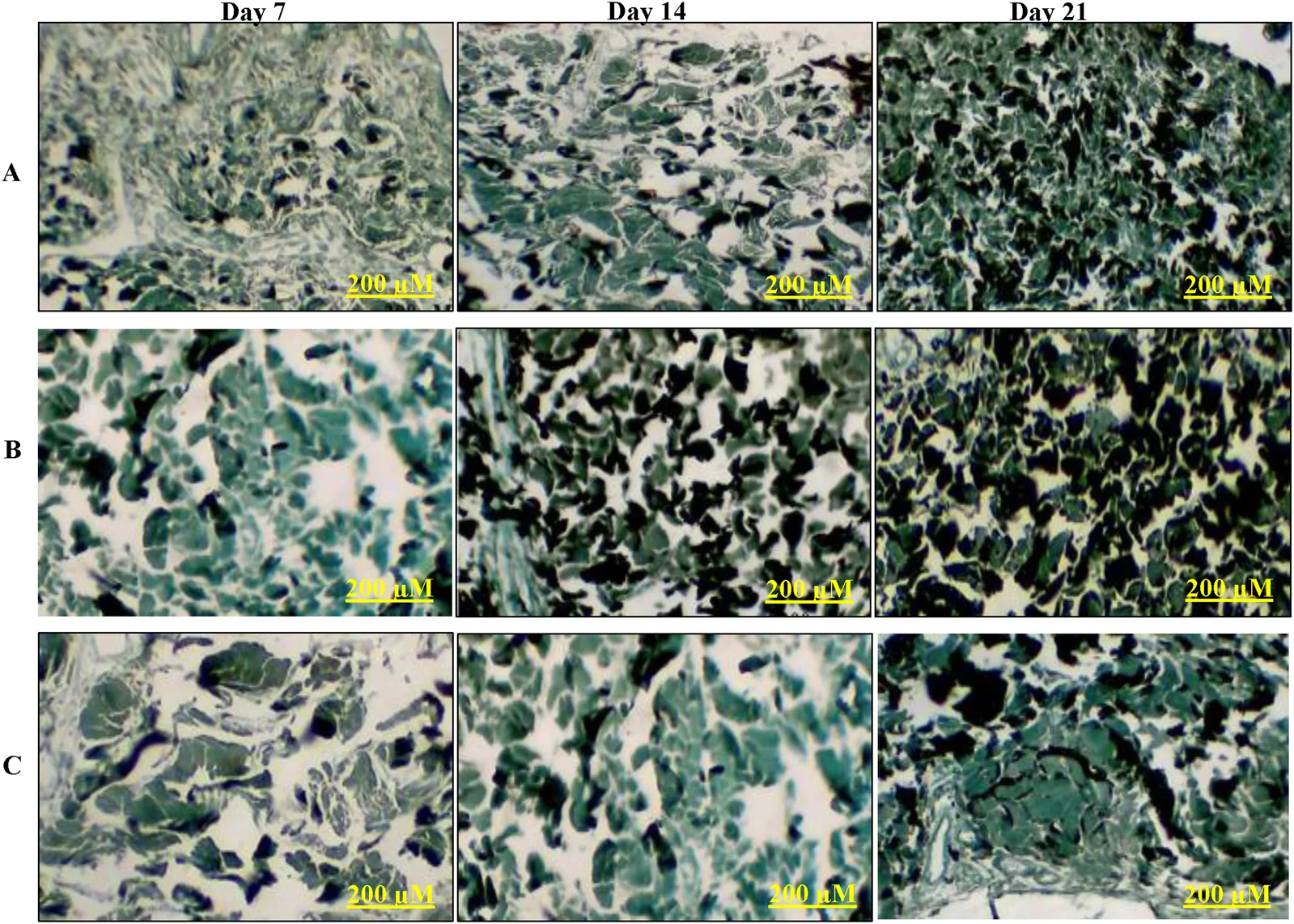
Masson’s trichrome staining of total collagen in Protocol A **(A)**, Protocol B **(B)**, and Protocol C **(C)** scaffolds following *ex vivo* incubation with fresh human tissue for 7, 14, and 21 days.

**FIGURE 18:**
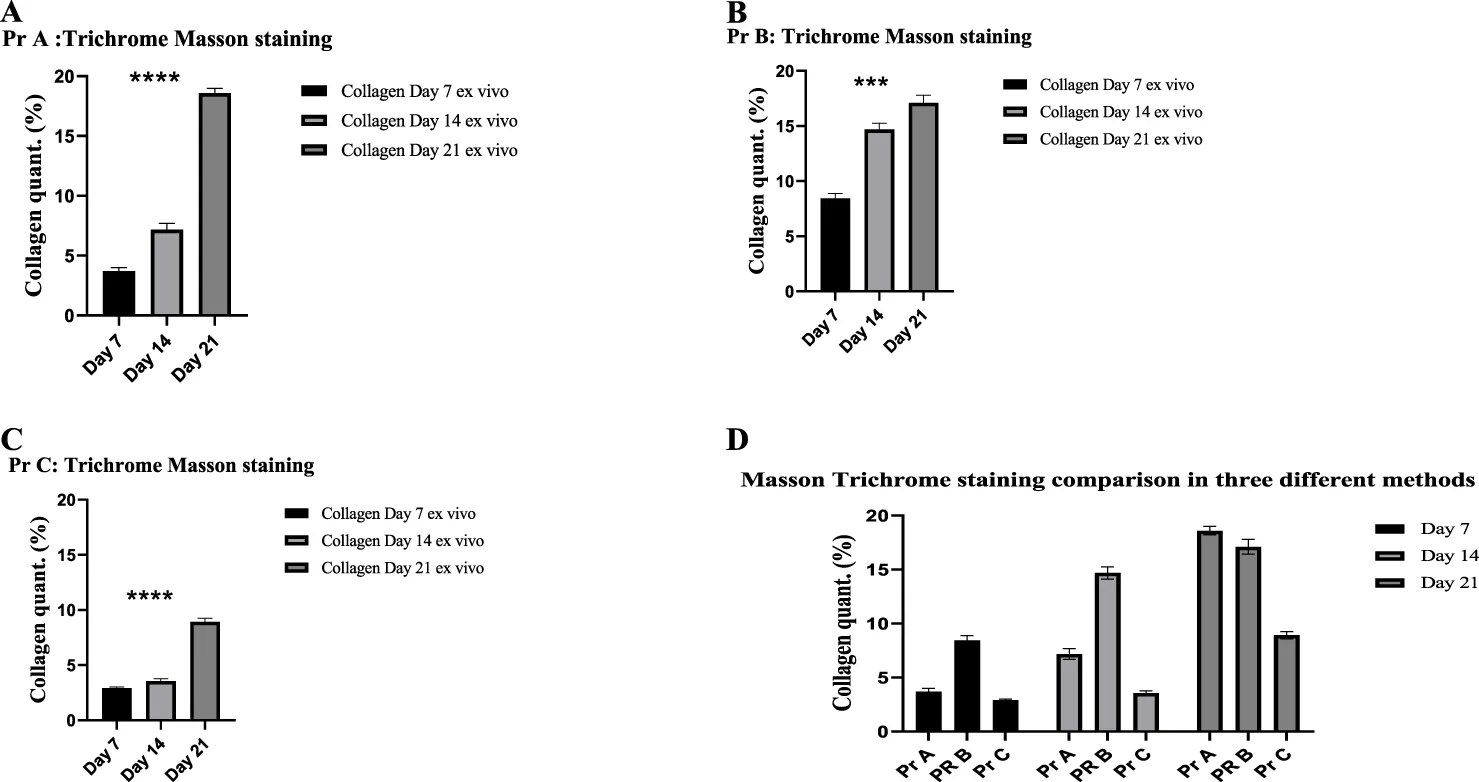
ImageJ quantification of total collagen in Protocol A **(A)**, Protocol B **(B)**, and Protocol C **(C)** scaffolds at days 7, 14, and 21. **(D)** Comparative quantification of total collagen across the three protocols and time points. Each protocol was performed three times (n = 3); data are presented as mean ± SD. **p < 0.01, ***p < 0.001, and ****p < 0.0001.

Total collagen content, assessed using Masson’s Trichrome staining, mirrored the trends observed with immunohistochemistry. Protocol B displayed the highest collagen accumulation, followed by Protocol A, whereas Protocol C showed the lowest accumulation (Figures 17A–C and 18A–C). Comparative analysis (Figure 18D) further highlighted Protocol B as the most effective in promoting ECM deposition and scaffold maturation. Overall, Protocol B promoted the greatest deposition of new collagen IV, elastin, laminin, and total collagen (Masson’s Trichrome) by day 21, confirming its superior ability to support active tissue remodeling in a human full-thickness skin environment (Figures 13–18). Collectively, these results demonstrate that low-detergent ADM scaffolds can actively integrate with native human tissue and support matrix remodeling consistent with regenerative activity *ex vivo*.

## 4 Discussion

The primary aim of this study was to optimize decellularization of human breast skin tissue to develop ADM scaffolds with enhanced properties for wound healing and tissue engineering. Two novel protocols (A and B), employing lower detergent concentrations combined with physical and enzymatic treatments, were compared with a previously published high-detergent method (Protocol C). This work was driven by the need to reconcile effective decellularization with preservation of ECM composition, ultrastructure, and biomechanical function which are increasingly recognized as critical determinants of scaffold performance in regenerative medicine (Badylak et al., 2009; Crapo et al., 2011; Gilbert et al., 2006). Our findings demonstrate that the optimized protocols, particularly Protocol B, achieved substantial cellular removal while preserving critical ECM proteins, maintaining tissue architecture, and supporting fibroblast proliferation. Aligning closely with prior studies demonstrating that milder decellularization strategies improve biological performance by limiting ECM disruption (Faulk et al., 2014; Keane et al., 2015).

This study distinguishes between technical and biological replicates, highlights donor-to-donor variability as a key source of experimental variation affecting scaffold outcomes, and notes that limited biological replication reduces statistical power and generalizability, underscoring the need for larger, better-matched donor cohorts in future work.

While Protocol A showed marginally superior performance in direct 2D fibroblast seeding assays (likely due to closer preservation of native fiber diameter and maximal ECM retention), Protocol B demonstrated substantially greater infiltration than both Protocols A and C in the *ex vivo* full-thickness wound model.

This divergence reflects fundamental differences between simplified *in vitro* cell–surface assays and biologically complex tissue environments that require active cell migration, three-dimensional infiltration, and dynamic matrix remodeling as previously highlighted in tissue engineering studies comparing simplified and physiologically relevant models (Badylak et al., 2009).

Protocol A, which relies primarily on osmotic lysis, preserved native collagen fiber diameter and maximized ECM retention, supporting robust cell attachment and proliferation under direct seeding conditions consistent with reports that preservation of native ECM ultrastructure enhances initial cell adhesion (Gilbert et al., 2006). In contrast, Protocol B incorporated a mild non-ionic detergent (0.1% Triton X-100), which likely facilitated partial loosening of interfibrillar packing and the creation of a more permeable microarchitecture, with similar effects of low-concentration detergents on increasing matrix accessibility without extensive protein loss reported in the literature (Faulk et al., 2014; White et al., 2017) (as inferred from SEM/topography and *ex vivo* ingress), though pore interconnectivity was not directly quantified. These subtle microstructural changes appear to enhance scaffold permeability, enabling more efficient cell ingress, spatial redistribution, and subsequent ECM deposition when cells must migrate from surrounding tissue rather than being artificially seeded onto the surface, in agreement with studies demonstrating the importance of porosity and interconnectivity for cell infiltration (Badylak et al., 2009; Faulk et al., 2014).

Mechanistically, this suggests that ultrasonication alone (Protocol A) is sufficient to enhance cellular clearance while conserving ultrastructure but may not consistently disrupt interfibrillar packing enough to increase pore interconnectivity at the scale required for rapid three-dimensional migration from wound edges consistent with reports that physical methods alone may preserve structure but have limited effects on permeability (Keane et al., 2015). The addition of 0.1% Triton X-100 in Protocol B likely lowers lipid-associated and weak protein–protein interactions and partially relaxes fiber bundling, *appears* to preserve a more open and interconnected ultrastructure based on qualitative SEM observations. Without the broad ECM solubilization associated with higher detergent exposure as described in studies on detergent-ECM interactions (White et al., 2017). This provides a plausible explanation for the marked divergence observed in the *ex vivo* model, where Protocol B reached ∼20,000 cells per 4-mm punch by day 21 *versus* ∼5,000 with Protocol A: the dominant limiting step is not surface adhesion, but access, i.e., the ability of host cells to enter, redistribute, and remodel the matrix in three dimensions consistent with previous scaffold integration studies (Badylak et al., 2009).

From a biophysical perspective, this suggests that optimal ADM performance depends not solely on maximal ECM preservation, but on achieving a balance between matrix integrity and pore interconnectivity that supports collective cell migration and mechanotransductive remodeling as emphasized in prior ECM scaffold literature (Crapo et al., 2011; Keane et al., 2015). This assay is therefore the most translationally relevant assay in the study, because it relies on cell migration from surrounding tissue and subsequent ECM remodeling, processes that better reflect clinical wound healing and scaffold integration as highlighted in previous regenerative medicine studies (Badylak et al., 2009).

The *ex vivo* findings therefore provide a more stringent and clinically informative evaluation of scaffold bifunctionality than direct cell-seeding assays alone (Figure 10 versus Figures 11–18) (Faulk et al., 2014; White et al., 2017).

This combined ultrasonication-low-detergent strategy directly addresses a central translational failure of many clinically available ADMs: adequate cellular clearance that nevertheless yields poor tissue integration due to excessive ECM compaction or loss of biological instruction, as previously reported in ECM scaffold studies (Badylak et al., 2009). By preserving basement membrane proteins while enhancing pore interconnectivity, Protocol B achieves a favorable balance between matrix fidelity and biological accessibility, a prerequisite for rapid scaffold integration in human wounds.

Efficient removal of cellular material is essential to minimize immunogenicity and inflammation as defined in established decellularization criteria (Crapo et al., 2011). All three protocols achieved greater than 90% reduction in DNA content, suggesting comparable decellularization performance under the current analytical conditions consistent with previous reported benchmarks (Keane et al., 2015). These findings suggest that differences in biological performance are more likely related to protocol-dependent effects on ECM preservation and scaffold microarchitecture than to major differences in residual cellular material, although this interpretation is limited by the absorbance-based DNA quantification method and absence of DNA fragment analysis. However, the key advantage of the optimized protocols lies in the preservation of ECM proteins. IHC and Raman spectroscopy revealed that Protocols A and B maintained higher levels of collagen IV, elastin, and laminin compared to Protocol C. Preservation of these ECM components is crucial because they mediate cell adhesion, migration, proliferation, and differentiation, directly influencing scaffold functionality as widely reported (Gilbert et al., 2006). Basement membrane proteins such as laminin and collagen IV are particularly important for guiding epithelial–mesenchymal interactions and re-epithelialization, while elastin contributes to mechanical resilience and long-term tissue compliance, in agreement with previous findings (Crapo et al., 2011). The superior ECM retention observed with Protocols A and B is likely attributable to reduced detergent concentration exposure combined with physical treatments such as ultrasonication, which aid cell removal while minimizing ECM disruption as reported in prior studies (Faulk et al., 2014; Keane et al., 2015). In contrast, the harsher chemical conditions in Protocol C caused significant ECM loss consistent with known detergent effects (White et al., 2017).

Structural analyses confirmed these findings. Masson’s Trichrome and Verhoeff’s Van Gieson staining demonstrated better collagen and elastin integrity in Protocol A, followed by Protocol B, while Protocol C showed notable collagen degradation and reduced elastic fiber content consistent with previous histological reports (Crapo et al., 2011; Gilbert et al., 2006). These histological differences aligned closely with the biochemical and functional outcomes observed across protocols. SEM analyses further indicated that Protocols A and B maintained scaffold morphology, porosity, and surface integrity, providing an optimal environment for cell attachment and proliferation as supported by previous studies (Badylak et al., 2009). In contrast, the larger and irregular collagen fiber diameter and appearance observed in Protocol C suggest disruption of the collagen network, potentially compromising mechanical integrity and cell-matrix interactions consistent with reports of detergent-induced ECM damage (White et al., 2017). Such alterations may impair mechano-transduction cues that are increasingly recognized as central regulators of fibroblast behavior and matrix remodeling.

Importantly, the key innovation of this study lies not in detergent reduction alone, but in the synergistic integration of controlled ultrasonication with minimal Triton X-100 exposure. Ultrasonication likely enhances decellularization efficiency by promoting convective transport and removal of cellular debris, while the low concentration of non-ionic detergent avoids widespread protein denaturation. Together, these processes appear to induce subtle microarchitectural reorganization, creating permissive micro-channels that enhance scaffold permeability and collective cell migration without eroding critical ECM signaling components.

Mechanical testing corroborated these structural observations. Protocols A and B produced scaffolds with higher ultimate tensile strength and stiffness (elastic modulus) compared to untreated and Protocol C samples, emphasizing that the optimized protocols preserve the mechanical integrity necessary to withstand physiological forces and support tissue regeneration consistent with reports of increased stiffness following decellularization (Keane et al., 2015). The increase in tensile strength that was observed in both Protocol A and B compared to native tissue, may be triggered by structural changes induced by decellularization. Decellularization may reduce tissue hydration and promote ECM densification, which enhances collagen fiber interactions and stiffness. Furthermore, cellular volume loss promotes ECM compaction and fiber alignment, increasing load-bearing efficiency. Finally, removal of cellular debris eliminates weak structural points and stress concentrators, resulting in a uniform collagen-rich scaffold. Similar alterations in mechanical behavior following decellularization have been reported previously in ECM scaffold studies (Badylak et al., 2009; Gilbert et al., 2006). In contrast, the reduced mechanical performance of Protocol C highlights the detrimental effects of excessive chemical exposure on scaffold robustness as previously reported (Faulk et al., 2014). The functional implication of increased elastic modulus requires careful interpretation. In dermal reconstruction, higher stiffness can be advantageous in the early phase by improving handling, suture retention, and resistance to deformation under load; however, excessive stiffening may also risk graft–host compliance mismatch, increasing strain gradients at the interface and potentially hindering integration in highly mobile anatomic sites. In this study, the improved *ex vivo* infiltration and ECM deposition observed with Protocol B suggest that the measured increase in stiffness did not impose a dominant compliance barrier under the tested conditions, but future *in vivo* work should explicitly evaluate interface mechanics to define the optimal stiffness “window” for clinical translation.

Functional assessment of the scaffolds using fibroblast cultures reinforced these structural and biochemical findings. Protocols A and B supported significant fibroblast proliferation and metabolic activity over 21 days, consistent with ECM preservation in the literature (Badylak et al., 2009), with Protocol A showing the highest performance in direct seeding assays. In contrast, Protocol C exhibited limited cell growth, likely due to ECM loss and disrupted scaffold architecture as previously described (Keane et al., 2015). However, in the *ex vivo* model, which more closely mimics clinical conditions, Protocol B supported markedly greater fibroblast infiltration, proliferation, and integration, resulting in substantially enhanced ECM deposition and scaffold maturation. This suggests that optimal scaffold performance depends not only on ECM retention but also on microstructural features that enable rapid host cell ingress and dynamic remodeling. Collectively, these findings indicate that optimized ADMs provide a favorable microenvironment for tissue regeneration.

Overall, the *ex vivo* analyses highlight the critical influence of decellularization protocol on scaffold integration capacity and functionality, in agreement with the current consensus in the field (Badylak et al., 2009; Crapo et al., 2011). Protocol B supported the highest cell numbers (∼20,000 by day 21) and ECM accumulation, followed by Protocol A (∼5,000 cells), while Protocol C showed minimal cellular integration and ECM remodeling (Figures 11–18). The consistency of these findings across histological, biochemical, mechanical, and functional endpoints strengthens the robustness of the conclusions. Therefore, although both novel protocols outperform the high-detergent control, Protocol B (0.1% Triton X-100 with ultrasonication) represents the most favorable balance between ECM preservation and support for rapid cell infiltration and tissue remodeling, supporting Protocol B as the strongest candidate for further translational evaluation. The concordance between H&E staining, IHC, and Masson’s Trichrome data confirms that Protocols A and B offer superior scaffold performance by preserving ECM composition, structure, and mechanical properties, all of which are essential for effective tissue engineering.

Despite these promising results, some limitations exist. The study was conducted on a limited sample size and a single tissue source (breast skin), which may limit generalizability to other skin types or tissues. Additionally, donor-to-donor variability and long-term scaffold remodeling beyond 21 days were not assessed. Future studies should investigate these protocols using larger sample sizes and diverse tissue sources. Additionally, *in vivo* studies are necessary to evaluate scaffold performance in clinically relevant wound healing and tissue regeneration models. Such studies will be essential to assess host immune responses, vascularization, and long-term functional integration.

From a clinical translation and manufacturing perspective, the use of controlled ultrasonication in combination with a minimal, widely accepted non-ionic detergent is particularly advantageous. This approach aligns with emerging trends toward scalable and regulatory-compatible decellularization strategies that minimize chemical exposure while preserving ECM integrity (Crapo et al., 2011). This approach avoids harsh chemistries, is readily scalable, and aligns with typical regulatory expectations for human-derived biomaterials (subject to defined residuals, bioburden/sterility assurance, and lot-to-lot consistency), positioning it as potentially adaptable to next-generation ADM production, pending validation of residual detergent levels, sterility, bioburden control, mechanical reproducibility, and lot-to-lot consistency. This strategy therefore has potential translational relevance beyond being a purely experimental refinement.

This study has several important limitations. A major limitation lies in the methodology used for DNA quantification. Residual DNA was measured using TRIzol extraction followed by absorbance-based detection, a method that lacks specificity for double-stranded DNA (dsDNA) and may overestimate DNA content due to contamination from RNA, proteins, or other residual biomolecules. Consequently, the reported residual DNA levels should be interpreted with caution. More accurate quantification would require the use of a dsDNA-specific fluorometric assay, such as the Qubit High Sensitivity assay.

In addition, DNA fragment size was not evaluated, preventing determination of whether residual DNA remained as intact or highly degraded fragments. This distinction is critical, as larger intact DNA fragments may pose a greater immunogenic risk than fragmented DNA. The limited sample size further reduced the statistical power of the study and may have limited the robustness and generalizability of the findings.

Furthermore, residual levels of Triton X-100, SDS, and Benzonase were not quantified, despite their known potential to influence cytocompatibility, inflammatory responses, and the overall biocompatibility and functional performance of decellularized scaffolds. Scaffold porosity and pore interconnectivity were also not quantitatively assessed, limiting comprehensive evaluation of the scaffold’s ultrastructural properties and its suitability for effective cell infiltration, nutrient diffusion, vascularization, and tissue integration.

Another important limitation is the absence of immune-related assays, such as macrophage polarization studies or cytokine profiling. Because the study was conducted using *ex vivo* models, the complex interactions of a functional immune system could not be replicated, thereby limiting interpretation of potential host inflammatory and immune responses. Consequently, *in vivo* studies are necessary to evaluate immunocompatibility, immunogenicity, and long-term host response to the decellularized scaffolds.

Future studies should incorporate dsDNA-specific quantification methods, DNA fragment size analysis, quantitative assessment of scaffold porosity and interconnectivity, and residual detergent/enzyme analysis to provide a more comprehensive characterization of scaffold quality and safety. Additionally, *in vivo* investigations combined with immune-response profiling will be essential to determine the translational potential and clinical applicability of the decellularized scaffolds.

In conclusion, this study demonstrates that low-detergent, sonication-assisted decellularization protocols, particularly Protocol B, can generate human ADM scaffolds with improved ECM preservation, structural integrity, mechanical performance, and cellular compatibility under the tested conditions. Protocol A remains a useful alternative where maximal retention of native-like ultrastructure is prioritized. Collectively, these findings support the rational development of human-derived dermal scaffolds and provide a foundation for further *in vivo* and translational evaluation.

## Data Availability

The data supporting the findings of this study are available from the corresponding author upon reasonable request due to privacy and confidentiality considerations.
